# Harnessing the Endogenous 2μ Plasmid of *Saccharomyces cerevisiae* for Pathway Construction

**DOI:** 10.3389/fmicb.2021.679665

**Published:** 2021-06-18

**Authors:** Jing Yang, Yujuan Tian, Huayi Liu, Yeyi Kan, Yi Zhou, Ying Wang, Yunzi Luo

**Affiliations:** ^1^Frontier Science Center for Synthetic Biology and Key Laboratory of Systems Bioengineering (Ministry of Education), School of Chemical Engineering and Technology, Collaborative Innovation Center of Chemical Science and Engineering (Tianjin), Tianjin University, Tianjin, China; ^2^Department of Gastroenterology, State Key Laboratory of Biotherapy, West China Hospital, Sichuan University, Chengdu, China

**Keywords:** 2μ plasmid, plasmid stability, tyrosol, pRS423, *Saccharomyces cerevisiae*

## Abstract

pRS episomal plasmids are widely used in *Saccharomyces cerevisiae*, owing to their easy genetic manipulations and high plasmid copy numbers (PCNs). Nevertheless, their broader application is hampered by the instability of the pRS plasmids. In this study, we designed an episomal plasmid based on the endogenous 2μ plasmid with both improved stability and increased PCN, naming it p2μM, a 2μ-modified plasmid. In the p2μM plasmid, an insertion site between the *REP1* promoter and *RAF1* promoter was identified, where the replication (ori) of *Escherichia coli* and a selection marker gene of *S. cerevisiae* were inserted. As a proof of concept, the tyrosol biosynthetic pathway was constructed in the p2μM plasmid and in a pRS plasmid (pRS423). As a result, the p2μM plasmid presented lower plasmid loss rate than that of pRS423. Furthermore, higher tyrosol titers were achieved in *S. cerevisiae* harboring p2μM plasmid carrying the tyrosol pathway-related genes. Our study provided an improved genetic manipulation tool in *S. cerevisiae* for metabolic engineering applications, which may be widely applied for valuable product biosynthesis in yeast.

## Introduction

Yeast, especially *Saccharomyces cerevisiae* (*S. cerevisiae*), has been developed as a host organism for the heterologous production of high-value compounds (Luo et al., 2015; Suastegui and Shao, 2016; Gao et al., 2017; Cao M. et al., 2020; Cao X. et al., 2020; Liu H. et al., 2020; Liu Q. et al., 2020; Ren et al., 2020), free fatty acid (Zhang et al., 2019), soluble cytosolic proteins (Boulet et al., 2017; González et al., 2018; Huang et al., 2018; Zhang et al., 2019), and biofuels (Zhang et al., 2021). Many genetic manipulations of *S. cerevisiae* rely on the utilization of plasmids (Romanos et al., 1992). There are three commonly used plasmids: (1) yeast-integrating plasmid (YIp) lacks the yeast replication initiation site and can only be stabilized when integrated into the yeast chromosome (Jensen et al., 2014). However, YIp brings only one copy of target sequences to the chromosome. (2) Yeast centromere plasmid (YCp) contains an autonomously replicating sequence (ARS) and a yeast centromere (CEN) (Chlebowicz-Śledziewska and Śledziewski, 1985; Lee et al., 2016), which has high mitotic stability but low copy number. (3) Yeast episomal plasmid (YEp) harbors a 2μ plasmid replication origin and a partitioning locus (*STB* or *REP3*) (Murray and Cesareni, 1986), which has high copy numbers but low stability (Hohnholz et al., 2017). In summary, plasmids with stable expression usually cannot provide high copy number, while plasmids with high copy number will be easily lost after long-term fermentation in the nutrient medium. Therefore, a stable plasmid system with high copy number is urgently needed.

Yeast endogenous 2μ plasmid is a cryptic nuclear plasmid (Stevens and Moustacchi, 1971; Petes and Williamson, 1975), which confers no phenotype beyond the ability to maintain itself a high copy number at 60–330 copies per cell with the help of FLP-mediated recombination (Gerbaud et al., 1979; Murray and Cesareni, 1986; Reider Apel et al., 2017). The 2μ plasmid is a circular DNA plasmid with a size of 6,318 bp and a circumference of about 2 μm (Hartley and Donelson, 1980).

In the 2μ plasmid, there is an ∼600-bp DNA sequence essential for the faithful partitioning of the 2μ plasmid along with the *trans-*acting ORFs *REP1* and *REP2* (Kikuchi, 1983), named *STB* (Murray and Cesareni, 1986). In the absence of *STB*, the 2μ-based plasmids are rapidly lost due to extreme mother bias during mitosis. In addition, the 2μ plasmid codes for four proteins (REP1, REP2, RAF1, and FLP) that are vital for its own survival. REP1 and REP2 are the primary factors responsible for the 2μ plasmid stability (Jayaram et al., 1983). RAF1 interacts with both REP1 and REP2 independently and blocks their interaction, thus reducing the cellular concentration of the REP1*–*REP2 complex that acts as a repressor of *REP1*, *FLP*, and *RAF1* genes. This blockage resulted in reduced plasmid stability and increased plasmid copy number (PCN). Both the deletion and overexpression of *RAF1* have a similar effect on the plasmid stability and copy number, resulting in an increased PCN and decreased plasmid stability (Rizvi et al., 2018). FLP is a conservative site-specific recombinase (Sadowski, 1995). The flip of one half of the 2μ plasmid with respect to the other is predominantly FLP dependent (Gerbaud et al., 1979; Broach and Hicks, 1980). The *FLP*-mediated recombination is also believed to be responsible for the interconversion of the plasmid replication between the theta and the rolling circle modes of replication.

Many researchers took advantage of the high PCN and stable inheritance of the 2μ plasmid to directly transform 2μ plasmid as an expression tool. Ludwig et al. selected the *HPA*I restriction site of *STB* as the insertion site (Ludwig and Bruschi, 1991), but the loss of *STB* led to a high loss rate of the plasmid (Murray and Szostak, 1983; McQuaid et al., 2019). Misumi et al. (2018) inserted the yeast promoter, terminator, and nutritional deficiency marker gene *leu2* between *RAF1* and *STB* and called this plasmid YHp. The application of YHp was restricted in [cir^0^] strains (Misumi et al., 2018). Zeng et al. (2021) chose two sites as the targets for insertion of heterogeneous DNA fragment: one is at the downstream of the *RAF1*, while the other is at the end of *REP2*. The derivative plasmids generated by inserting the same target gene at these two sites have lower plasmid loss rates and better expression level than the conventional 2μ-based plasmid pRS425 (Zeng et al., 2021). To our knowledge, no commonly used methods have been developed in laboratory strains with the wild-type (WT) 2μ plasmid (Supplementary Figure 1A).

Based on these previous studies described above (Hartley and Donelson, 1980; Jayaram et al., 1983; Rizvi et al., 2018; McQuaid et al., 2019), we identified a new insertion site between the *REP1* promoter and *RAF1* promoter (Supplementary Figure 1B). The pBR322ori, *KanMX* selection marker gene, and three endonuclease sites *Xho*I/*Pme*I/*Not*I were inserted in this site. The 2μ-modified plasmid was named p2μM. In plasmid stability measurement, the p2μM plasmid system was more stable than the pRS423 plasmid system. To test the application of p2μM in the biosynthesis of natural products, the tyrosol [a phenethyl alcohol derivative that has antioxidant and anti-inflammatory effects (Choe et al., 2012)] pathway-related genes were introduced into p2μM. The results confirmed that the stability and property of the p2μM were better than those of the pRS423M plasmid. Our study provided an improved genetic manipulation tool in *S. cerevisiae* for metabolic engineering applications, and it may be widely applied in valuable natural product biosynthesis in yeast.

## Design and Construction of Endogenous 2μ-Based Plasmids *In Vitro*

In order to construct a stable endogenous 2μ-based plasmid and apply it for DNA expression and pathway construction, the proper insertion site should be selected to insert essential elements and heterogeneous DNA fragments. Besides the known genes and sequences, there are still uncharacterized transcripts transcribed from the 2μ plasmid (Rizvi et al., 2017). It was found that the promoters of *RAF1* and *REP1* on the endogenous 2μ plasmid were adjacent and there was no other element between them by analyzing the elements related to stability. Thus, this site was selected as the insertion site (Supplementary Figure 1A). To edit the endogenous 2μ plasmid for a better genetic manipulation tool, the origin replication of *Escherichia coli*, combined with G418 resistance marker, was chosen to be inserted to construct p2μM (Supplementary Figure 1B).

To characterize the property of the p2μM plasmid, plasmid pRS423 with G418 resistance was chosen as a control to generate plasmid pRS423M (Supplementary Figure 1C). Plasmid pRS423 is also commonly used in yeast among the YEp pRS42 series plasmids due to its relatively high stability and copy number (Christianson et al., 1992).

Tyrosol is mainly extracted from olive oil, wine, and plant tissues. It has proven to be an effective cellular antioxidant and is widely used in food and medicine industries (Benedetto et al., 2007; Karković Marković et al., 2019). Taking into account the impact of the size of inserted fragment on the p2μM plasmid, we constructed three modules of different sizes using genes of the tyrosol biosynthetic pathway (Supplementary Figure 1D). The small module (mutation module, 3.8 kb) of *ARO4^K229L^* and *ARO7^G141S^* could efficiently relieve feedback inhibition and increase the production of tyrosol in *S. cerevisiae* (Liu H. et al., 2020), which was introduced to generate plasmid p2μM-*ARO4^K229L^-ARO7^G141S^* (p2μM-small-module). The rewiring module containing pentose phosphate pathway genes *TKL1* and *RKI1* could tune the flux of the precursor pathway (Walfridsson et al., 1996; Kondo et al., 2004; Bera et al., 2011). The adjustment module that contains *ARO2* and *ARO10* could adjust the shikimate pathway and L-tyrosine branch by catalyzing the conversion of chorismate from EPSP and the decarboxylation of 4-HPP to 4-HPPA (Liu H. et al., 2020), respectively. The medium module (9.8 kb) composed of the rewiring module and the adjustment module was overexpressed by p2μM plasmid, resulting in plasmid p2μM-*TKL1-RKI1-ARO10-ARO2* (p2μM-medium-module). Finally, the medium module was introduced into plasmid p2μ-small-module, resulting in plasmid p2μM-*TKL1-RKI1-ARO10-ARO2-ARO4^K229L^-ARO7^G141^*S (p2μM-large-module, the size of the large module was 13.6 kb). Then, these three modules were also inserted into the multiple cloning sites of plasmid pRS423M to generate pRS423M-small-module, pRS423M-medium-module, and pRS423M-large-module, collectively called pRS423M-based plasmids (Supplementary Figure 1F). The structures of the three modules are shown in Supplementary Figure 2.

## Determination of Plasmid Stability

**FIGURE 1:**
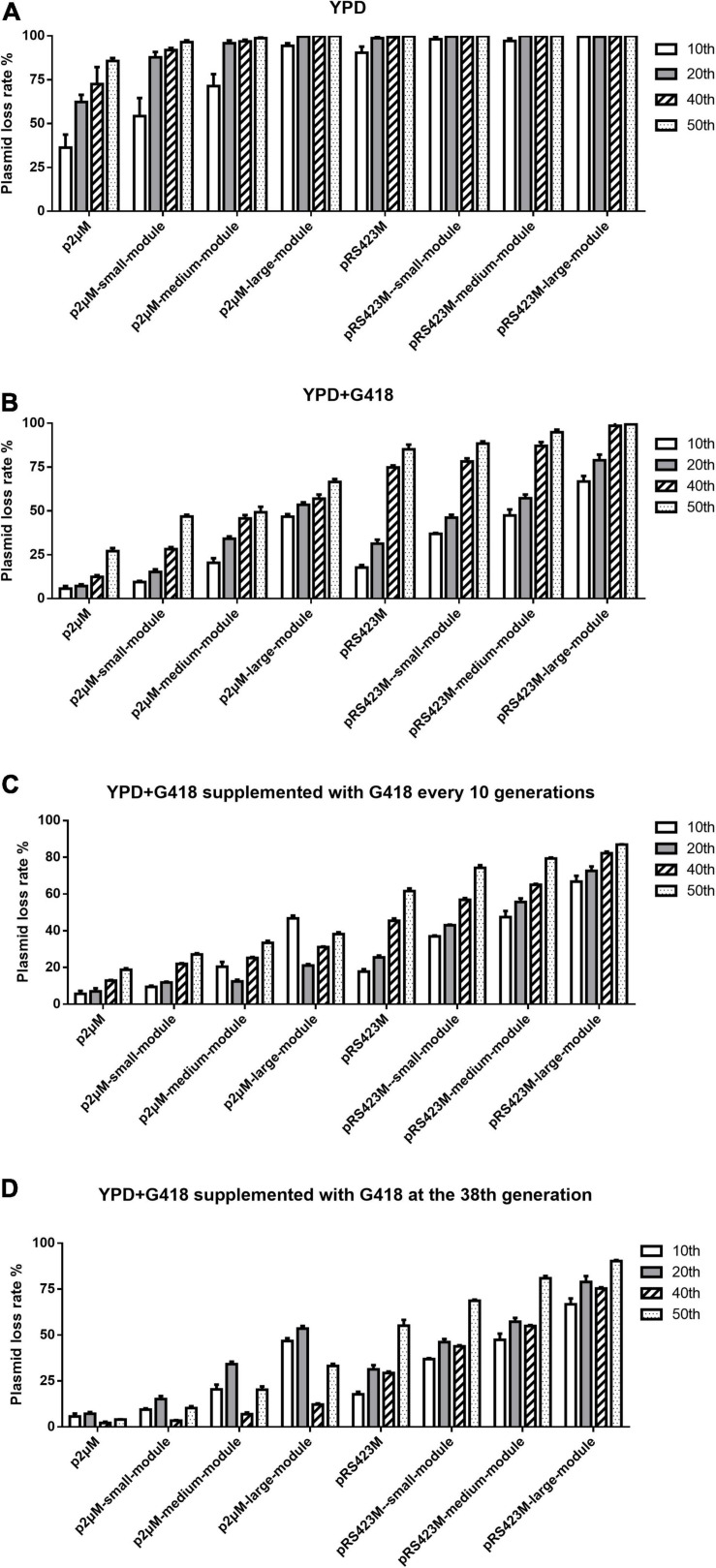
Plasmid loss rates of the p2μM-based plasmids with different sizes of inserted fragments compared with that of pRS423M-based plasmids. **(A)** Plasmid loss rates of the p2μM-based plasmids and the pRS423M-based plasmids after cultivation in YPD medium. **(B)** Plasmid loss rates of the p2μM-based plasmids and the pRS423M-based plasmids after cultivation in YPD + G418 medium. **(C)** Plasmid loss rates of p2μM-based plasmids and pRS423M-based plasmids in YPD + G418 medium supplemented with G418 every 10 generations. **(D)** Plasmid loss rates of p2μM-based plasmids and pRS423M-based plasmids in YPD + G418 medium supplemented with G418 at the 38th generation.

Since the yeast endogenous 2μ plasmid showed high stability and copy number, we assumed that our p2μM plasmid could be more stable than the pRS423M plasmid. To test this hypothesis, the influences of the size of the inserted fragment on the stability of the p2μM plasmid were explored *via* measuring the plasmid loss rate. As shown in Supplementary Tables 1, 2, the stabilities of the p2μM-based plasmids were significantly higher than those of the pRS423M-based plasmids. First, plasmid p2μM and pRS423M were transformed to *S. cerevisiae* strain CEN.PK2-1C, respectively. Then, the plasmid loss rates of the 10th, 20th, 40th, and 50th generation strains were tested in YPD without G418 and in YPD + G418 medium (Figures 1A,B). When the size of the inserted fragment was 0, the plasmid loss rates of plasmid p2μM in non-selective medium were 36.3 ± 6.0% for the 10th generation, 62.4 ± 3.3% for the 20th generation, 72.5 ± 7.9% for the 40th generation, and 85.7 ± 1.4% for the 50th generation, lower than those of the pRS423M plasmid (90.4 ± 2.9, 98.8 ± 0.9, 99.3 ± 0.2, and 99.9 ± 0.2%). Plasmid loss rates of p2μM in selective medium were 5.7 ± 1.3, 7.2 ± 0.7, 12.4 ± 0.8, and 27.1 ± 1.4% for each generation, which were much lower than those of pRS423M (17.8 ± 1.1, 31.4 ± 1.8, 74.8 ± 0.9, and 85.1 ± 2.2%).

Furthermore, three p2μM-based plasmids of the experimental group and three pRS423M-based plasmids of the control group mentioned above were transformed to strain CEN.PK2-1C, respectively. The results showed that the stabilities of p2μM-based plasmids were higher than those of pRS423M-based plasmids both in non-selective medium and selective medium (Figures 1A,B). For non-selective medium, when the sizes of the inserted fragments were 3,842 and 9,821 bp, the plasmid loss rates of p2μM-based plasmids were 54.3 ± 8.5 and 71.4 ± 5.6% (the 10th generation), 87.9 ± 2.4 and 95.8 ± 1.3% (the 20th generation), 91.9 ± 1.0 and 96.9 ± 0.8% (the 40th generation), and 96.4 ± 0.9 and 98.7 ± 0.3% (the 50th generation), while the plasmid loss rates of pRS423M-based plasmids were 98.1 ± 1.3 and 97.2 ± 1.1% for the 10th generation, and the plasmids were all lost at the 20th generation (99.7 ± 0.4 and 100.0%). Until the size of the inserted fragment increased to about 14 kb, the plasmid loss rate of the experimental group was 94.3 ± 1.2% for the 10th generation, but plasmids of the control group were almost all lost. For cultures that were grown in selective medium, when the fragment of 9,821 bp was introduced, 49.3 ± 2.5% strains lost their plasmid p2μM-medium-module, but almost all strains lost the plasmid pRS423M-medium-module after fermentation for 50 generations (94.9 ± 1.3%). All strains lost the plasmid pRS423M-large-module at the 40th generation (98.6 ± 1.2%); however, the plasmid loss rate of the p2μM-large-module was merely 57.0 ± 1.9%. The amounts of plasmid loss in YPD + G418 medium were less than those in YPD medium without G418.

As shown in Figure 1C, supplementing antibiotics to YPD + G418 medium every 10 generations could maintain lower plasmid loss rates. Plasmid loss rates of the 40th generation were greatly decreased after G418 was supplemented at the 38th generation (Figure 1D). The plasmid loss rates of the 40th generation were lower than those of the 20th generation, and the plasmid loss rates of p2μ-derived plasmids were still much lower than those of pRS423-derived plasmids.

## Plasmid p2μM Applied in Tyrosol Production

To demonstrate that p2μM could be applied for the optimization of natural product biosynthesis, the tyrosol biosynthetic pathway was chosen as an example. The WT strain CEN.PK2-1C was fermented in YPD medium. Engineered strains containing individual p2μM-based plasmids and pRS423M-based plasmids with different sizes of tyrosol biosynthesis-related modules were simultaneously fermented in both non-selective medium and selective medium.

**FIGURE 2:**
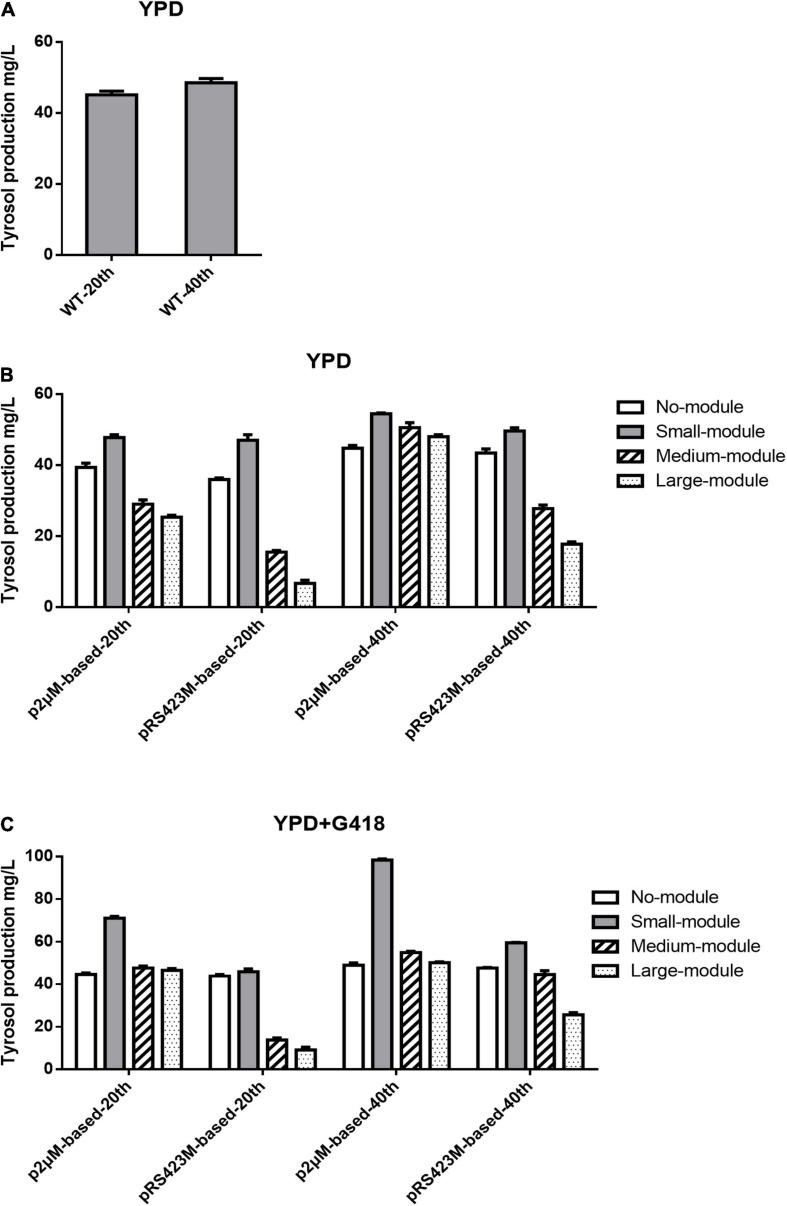
Tyrosol production of strains containing a single plasmid with modules of different sizes after fermentation for 20 and 40 generations. **(A)** Tyrosol production of the WT strain CEN.PK2-1C. **(B)** Tyrosol production of strains CEN.PK2-1C with p2μM-based plasmids and CEN.PK2-1C with pRS423M-based plasmids after fermentation in YPD medium. **(C)** Tyrosol production of strains CEN.PK2-1C with p2μM-based plasmids and CEN.PK2-1C with pRS423M-based plasmids after fermentation in YPD medium with 200 μg/ml G418.

As demonstrated in Figure 2A, after fermentation in YPD medium, tyrosol productions of the WT strain were 45.11 ± 0.85 mg/L at the 20th generation and 48.53 ± 0.98 mg/L at the 40th generation. In non-selective YPD medium, strain CEN.PK2-1C with p2μM produced 39.39 ± 0.97 mg/L tyrosol after 20 generations and 44.78 ± 0.64 mg/L tyrosol after 40 generations (Figure 2B), which were lower than those of the WT strain. When the plasmid p2μM-small-module was transformed into the strain CEN.PK2-1C, the tyrosol production was 47.79 ± 0.64 mg/L at the 20th generation and 54.46 ± 0.21 mg/L at the 40th generation, 12.2% greater than that of the WT strain and 9.7% greater than that of the strain with pRS423M-small-module. The strain CEN.PK2-1C carrying plasmid p2μM-medium-module accumulated 50.59 ± 1.12 mg/L tyrosol after 40 generations of fermentation. In the strain CEN.PK2-1C with p2μM-large-module, the tyrosol titer of 48.03 ± 0.45 mg/L was obtained, which was not as good as the WT strain but 7.3% higher than that of CEN.PK2-1C carrying p2μM. CEN.PK2-1C carrying plasmid pRS423M produced 35.99 ± 0.35 mg/L tyrosol at the 20th generation and 43.41 ± 0.94 mg/L tyrosol at the 40th generation, which were lower than those of the strain with p2μM and the WT strain. Tyrosol productions in strain CEN.PK2-1C with pRS423M-medium-module and pRS423M-large-module at each generation were all much lower than those of the strains carrying p2μM -based plasmids.

According to Figure 2C, after shake flask cultivation in YPD + G418 medium, the strain harboring p2μM generated tyrosol titer of 44.75 ± 0.83 mg/L at the 20th generation. At the 40th generation, tyrosol production was 49.05 ± 0.90 mg/L, which was higher than that of the WT strain and CEN.PK2-1C with p2μM fermented in non-selective medium; 71.11 ± 0.71 and 98.39 ± 0.41 mg/L tyrosol was produced in the strain containing p2μM-small-module after fermentation for 20 and 40 generations, respectively, which were much higher than that of CEN.PK2-1C with pRS423M-small-module (59.55 ± 0.16 mg/L). Tyrosol productions accumulated in the strain with p2μM-medium-module (47.71 ± 0.72 and 54.95 ± 0.50 mg/L) and p2μM-large-module (46.44 ± 0.65 and 50.20 ± 0.34 mg/L) after fermentation for 20 and 40 generations in selective medium were lower than those of the strain containing p2μM-small-module, but they were higher than those of CEN.PK2-1C with pRS423M-based plasmids. Strains carrying pRS423M produced 47.72 ± 0.18 mg/L tyrosol at the 40th generation, 2.8% lower than that of the strain with p2μM and 1.7% lower than that of the WT strain. The tyrosol yields of the strain containing plasmids pRS423M-small-module (59.55 ± 0.13 mg/L), pRS423M-medium-module (44.65 ± 1.46 mg/L), and pRS423M-large-module (25.64 ± 0.80 mg/L) at the 40th generation were all lower than those of the strains of p2μM-based plasmids with modules of the same size.

All results showed that the tyrosol yields of the strains with p2μM -based plasmids were higher than those of the strains with pRS423M-based plasmids both in non-selective medium and selective medium, which could be due to the instability of plasmid pRS423.

## Discussion

In this study, an endogenous 2μ-based expression vector with enhanced stability was developed in *S. cerevisiae*. The site between the *RAF1* promoter and *REP1* promoter on this plasmid was chosen as the insertion site for the gene of interest, which would not affect the functional elements and stability of the plasmid.

The plasmid loss rates were calculated on the strains harboring plasmids with inserted fragments of different sizes by culturing in non-selective YPD medium and YPD medium with selective pressure. After culturing without selective pressure for 40 generations, the loss rates of p2μM and pRS423M were about 73 and 100%, respectively. For plasmids containing modules of about 4 kb, the plasmid loss rates of p2μM-small-module and pRS423M-small-module in non-selective YPD medium were about 90 and 100%, respectively. All strains lost their plasmids by fermentation in YPD medium for 50 generations. Culturing in YPD + G418 medium for 50 generations, plasmid loss rate of p2μM was about 27% and that of pRS423M was about 85%. Plasmid pRS423M-large-module was all lost after 40 generations of cultivation, while merely 57% of the plasmid p2μM-large-module was lost. Continuous supplementation of G418 in YPD + G418 medium could help maintain the stability of plasmids, especially for p2μM-based plasmids. The plasmid loss rate of p2μM-large-module after 40 generations of cultivation was about 31%, which was much lower than that of pRS423M-large-module (about 82%). Although the selection pressure was conducive to the stable existence and inheritance of plasmids, a large number of pRS423M-based plasmids were lost during long-time fermentation. The results showed that the stabilities of the p2μM-based plasmids were higher than those of the pRS423M-based plasmids. It is estimated that an inserted fragment of 10 kb is acceptable for p2μM when there is no selection in the medium, and the inserted fragment of 14 kb is acceptable for p2μM under condition with selection. Zeng et al. (2021) moved the essential gene *TPI1* from chromosome to p2μ plasmid. With auxotrophic complementation of *TPI1*, the resulting plasmid pE2μRT could undergo cultivation of 90 generations without loss under non-selective conditions.

Tyrosol biosynthetic pathway was introduced to demonstrate that the expression level of the p2μM-based plasmids was superior to that of the controls. After 40 generations of shake flask cultivation in YPD medium, the tyrosol yield of strain CEN.PK2-1C carrying plasmid p2μM-small-module was 54.46 ± 0.21 mg/L, about 9.7% higher than that of CEN.PK2-1C with pRS423M-small-module (49.64 ± 0.71 mg/L). The tyrosol titer of CEN.PK2-1C with p2μM-medium-module was 82.0% higher than that of strains carrying pRS423M-medium-module. The yield of tyrosol harvested from strains with p2μM-large-module was about threefold higher than that from strains with pRS423M-large-module. However, strains containing large module accumulated less tyrosol than strains containing small module and medium module, which was probably due to the instability of p2μM containing large module. Tyrosol production of the strain with p2μM-small-module at the 40th generation was 98.39 ± 0.41 mg/L with selective pressure, which was 80.7% greater than the strain with p2μM-small-module in non-selective medium and 65.2% higher than that of the strain with pRS423M-small-module in selective medium. The tyrosol yields of the strain containing plasmids pRS423M-medium-module and pRS423M-large-module at the 40th generation were all lower than those of the strains of p2μM-based plasmids with modules of the same size.

Taking these results into account, in order to improve the stability of endogenous 2μ-based expression vector in yeast, an essential gene could be introduced into the plasmid while knocking out the same essential gene in the genome to ensure the existence of engineered endogenous 2μ plasmid in yeast (Zeng et al., 2021). In the future, researchers could apply the CRISPR/Cas9 system to directly integrate metabolic pathways into the endogenous 2μ plasmid with an essential gene *in vivo* (Dean-Johnson and Henry, 1989; Zheng et al., 1993; Wang et al., 2020; Yang et al., 2021). In summary, our endogenous 2μ-based expression vector p2μM has improved stability than the commonly used YEp pRS423, so it could be applied in *S. cerevisiae* for genetic manipulations.

## Data Availability Statement

The original contributions presented in the study are included in the article/Supplementary Material, further inquiries can be directed to the corresponding author/s.

## Author Contributions

YT, JY, and YL conceived the study and carried out the molecular genetic studies as well as the strain construction. HL, YK, YZ, and YW participated in the design and coordination of the study. YT and JY performed the experiments and drafted the manuscript. YL supervised the whole research and revised the manuscript. All the authors read and approved the final manuscript.

## Conflict of Interest

The authors declare that the research was conducted in the absence of any commercial or financial relationships that could be construed as a potential conflict of interest.
