# Supplementary material for: Harnessing the Endogenous 2μ Plasmid of Saccharomyces cerevisiae for Pathway Construction
Source: Front Microbiol. 2021 Jun 18;12:679665. doi: 10.3389/fmicb.2021.679665 (PMC8249740; doi:10.3389/fmicb.2021.679665)
Supplement: Supplementary file 1 [file Data_Sheet_1.docx]

Supplementary Material

# 1 Materials and Methods

## 1.1 Strains and growth conditions

All strains used in this work were based on *Saccharomyces cerevisiae* CEN.PK2-1C and BY4741α, which were listed in **Supplementary** **Table 3**. Strain CEN.PK2-1C was used for genetic manipulations and *E. coil* strains were used for sub-cloning of plasmids. All the plasmids used in this study were listed in **Supplementary** **Table 4**. Cultures of *S. cerevisiae* strains were grown at 30 °C in YPD medium containing 20 g/L Peptone, bacteriological (Sangon Biotech﹟A100636), 10 g/L Yeast Extract and 20 g/L Dextrose with 200 μg/ml G418 if necessary (Hadfield et al., 1990; Si et al., 2014). The *E. coli* strains were grown at 37 °C in Luria-Bertani (LB) medium (10 g/L Tryptone, 5 g/L Yeast Extract and 10 g/L NaCl) with 50 μg/ml Kanamycin if necessary.

## 1.2 Construction of plasmids

Plasmids constructed in this study were listed in **Supplementary Table 4** All the fragments were amplified by PCR using the primers described in **Supplementary** **Table 5**. The wild-type genes (*TKL1*, *RKI1*, *ARO2* and *ARO10*) and the mutation genes *ARO4^K229L^* and *ARO7^G141S^* were amplified from strains constructed previously in our lab (Liu et al., 2020a). All promoters and terminators were amplified from the *S. cerevisiae* BY4741α except the original promoters and terminators, such as *ARO4*-promoter, *ARO4*-terminator, *ARO7*-promoter and *ARO7*-terminator, which were amplified from *S. cerevisiae* CEN.PK2-1C. The final expression plasmids and donor vectors were all constructed by the Gibson assembly method (Gibson et al., 2009). Site-directed mutagenesis was used to generate mutations and confirmed by Sanger sequencing.

Four p2μM plasmids were constructed based on the endogenous 2μ plasmid from *S. cerevisiae*. The 2μ plasmid was divided into two fragments by PCR using primers F1-2μ (*REP2*+30a) and R1-2μ (*REF1*p+41a), primers F2-2μ (*RAF1*p+48a) and R2-2μ (*REP2*+28a), respectively. pBR322 ori was amplified from pCas by PCR using primers F3-pBR322+26a and R3-pBR 322 ori+30a. *TEF1*p-*KanMX*-*TEF2*t was amplified from pCas by PCR using primers F4-*TEF1*p+51a and R4-*TEF2*t+54a. Then two DNA fragments were assembled together by overlap extension PCR (OE-PCR). All DNA fragments were also assembled together by over-lap extension PCR (OE-PCR) to construct p2μM. Modules and vectors were digested by appropriate restriction endonuclease and ligated together by T4 ligase. Four pRS423-based plasmids were constructed in a similar way.

Briefly, yeast colonies were picked and suspended in 20 μl of 20 mmol/L NaOH, followed by four cycles (95 °C for 4 min, 4 °C for 90 s) incubation in a thermocycler using PCR with KOD FX PCR mix (Toyobo, Osaka, Japan), which were then verified by DNA sequencing. The verified positive colonies were cultured in a test tube containing YPD+G418 medium for 18 hours, and then stored in 25% glycerol solution at -80 °C. For propagation, the verified positive colonies were cultured at 30 °C for 48 hours.

## 1.3 Plasmid stability measurement

To measure the stability of the p2μM-based plasmids and the pRS423M-based plasmids, strains were grown in non-selective medium and selective medium. At least three single colonies containing individual plasmids were picked from YPD+G418 medium plates and inoculated to separate vials containing 4 ml non-selective YPD medium. Three single colonies were inoculated to separate vials containing 4 ml YPD+G418 medium. These cultures were set as at 0 generation and were incubated at 30 °C for 20 h to obtain cultures of the 10th generation. For strains that grown in non-selective medium, 1 μl of the cultures was re-inoculated to 4 ml non-selective YPD medium and cultured another 20 h to obtain cultures of the 20th generation, then serial sub-cultures were conducted to obtain cultures of the 40th and the 50th generation. For strains that grown in YPD+G418 medium, the cultures were not re-inoculated but continuously cultivated to each generation. Three of the cultures with G418 were supplemented with 200 μg/ml G418 every 10 generations, and cultured continuously to the 50th generation. Another three cultures with G418 were supplemented with 200 μg/ml G418 at the 38th generation, and then continued to be cultured to the 50th generation. The cultures of each generation were diluted and plated equally onto several non-selective YPD and selective YPD+G418 plates. After 2 days growth, colonies were counted to calculate the plasmid loss rate. The OD_600_ values of the cultures were measured every 2 generations (every 4 hours).

## 1.4 Cultivations and fermentations

The single colonies were picked from pre-cultured plates and inoculated in 4 ml YPD medium at 30 °C, 220 rpm for 18 h. Then portions of the seed culture were transferred into 50 ml YPD medium in 250 ml shake flasks. Other portions of the seed culture were transferred into 50 ml YPD medium with 200 μg/ml G418. The OD_600_ was about 0.2. The strains were cultured at 30 °C for 100 h, shaking at 220 rpm. To monitor the cell growth, the samples fermented for different time were measured at 600 nm wavelength with a spectrophotometer. 500 μl samples were collected at the 20th and 40th generation. Centrifuged at 5000 rpm for 5 min, the supernatant was stored at -20 °C until analyzed.

## 1.5 Analytical method of tyrosol production

The samples were analyzed using an Shimadzu HPLC LC-20A series instrument equipped with a Chromplus-C18 column (SWELL, 4.6 mm × 150 mm, 5 μm), which was maintained at 30°C (Liu et al., 2020a). Tyrosol was detected by absorbance at 224 nm. Mobile phase A was 0.05% formic acid in water and solvent B was acetonitrile. The system was operated at a flow of 1 ml/min, with the following gradient: 0-18 min 8% B, then 5% B from 18.01 to 20 min, linearly increased to 100% B (20-30 min), held 95% B for 5 min, finally decreased to 8% B for 15 min. Standard curves were generated with four known concentrations of the tyrosol standards (Solarbio, China) dissolved in culture medium and were used for metabolite quantification. The R^2^ coefficient for the calibration curve was higher than 0.999. At least three biological replicates were analyzed for each strain.

# 2 Supplementary Figures and Tables

## 2.1 Supplementary figures


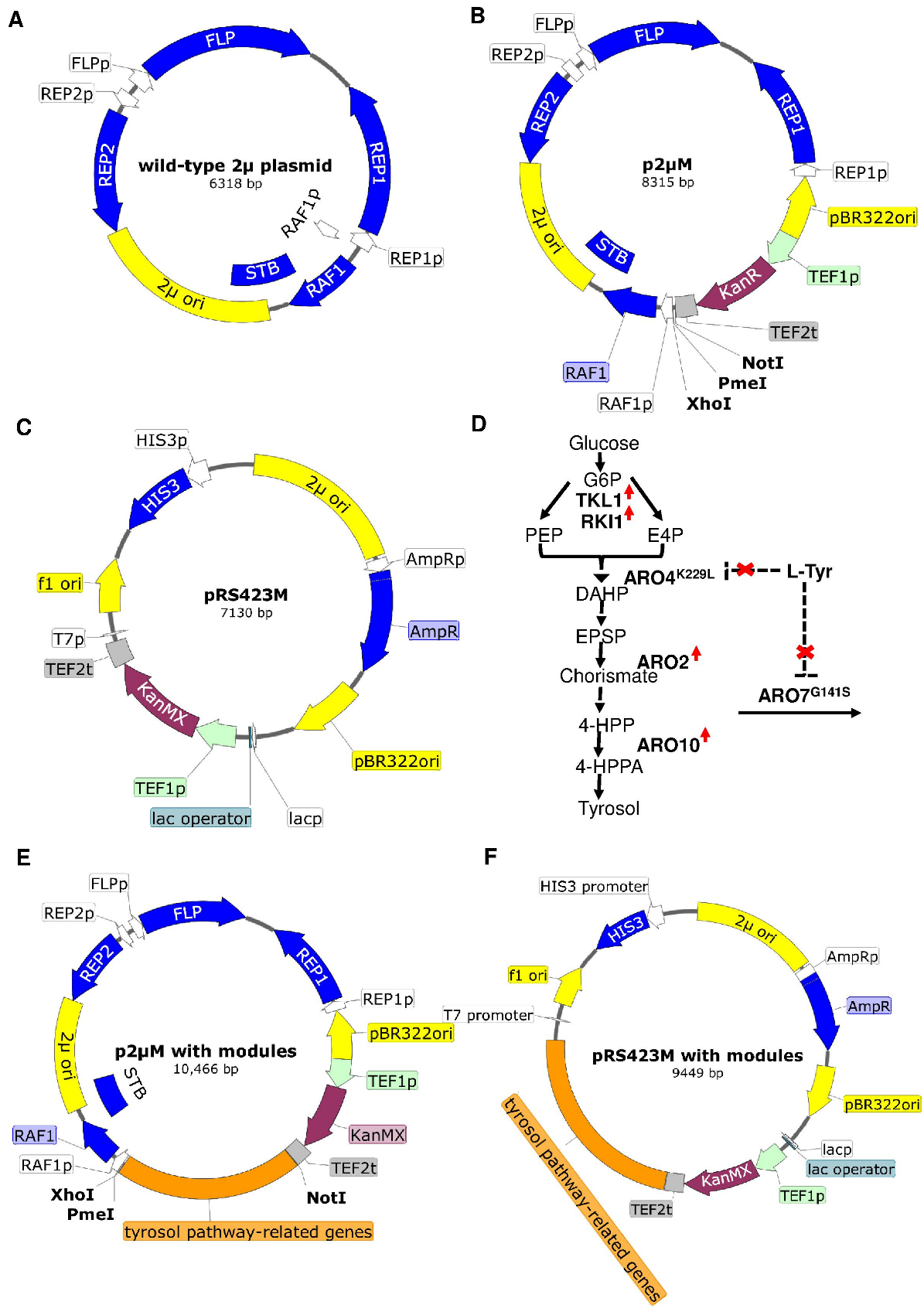


**Supplementary Figure 1.** The design of endogenous 2μ-based plasmids and pRS423-based plasmids with tyrosol biosynthetic pathway in vitro. **(A)** Wild type 2μ plasmid with no selective marker. **(B)** The p2μM plasmid: 2μ plasmid containing pBR322ori and KanMX. **(C)** The pRS423M plasmid containing KanMX. **(D)** Optimized tyrosol biosynthetic pathway in S. cerevisiae. Metabolite abbreviations: G6P: Glucose 6-phosphate; PEP: phosphoenolpyruvate; E4P: D-Erythrose 4-phosphate; DAHP: 3-deoxy-D-arabino-heptulosonate-7-phosphate; EPSP: 5-enolpyruvyl-3-shikimate; 4-HPP: p-hydroxyphenylpyruvate; 4HPAA: 4-hydroxyphenylacetylaldehyde. **(E)** The construction of p2μM-based plasmids with tyrosol pathway-related genes. The tyrosol biosynthetic pathway is shown in orange. **(F)** The construction of pRS423M-based plasmids containing tyrosol biosynthetic pathway.


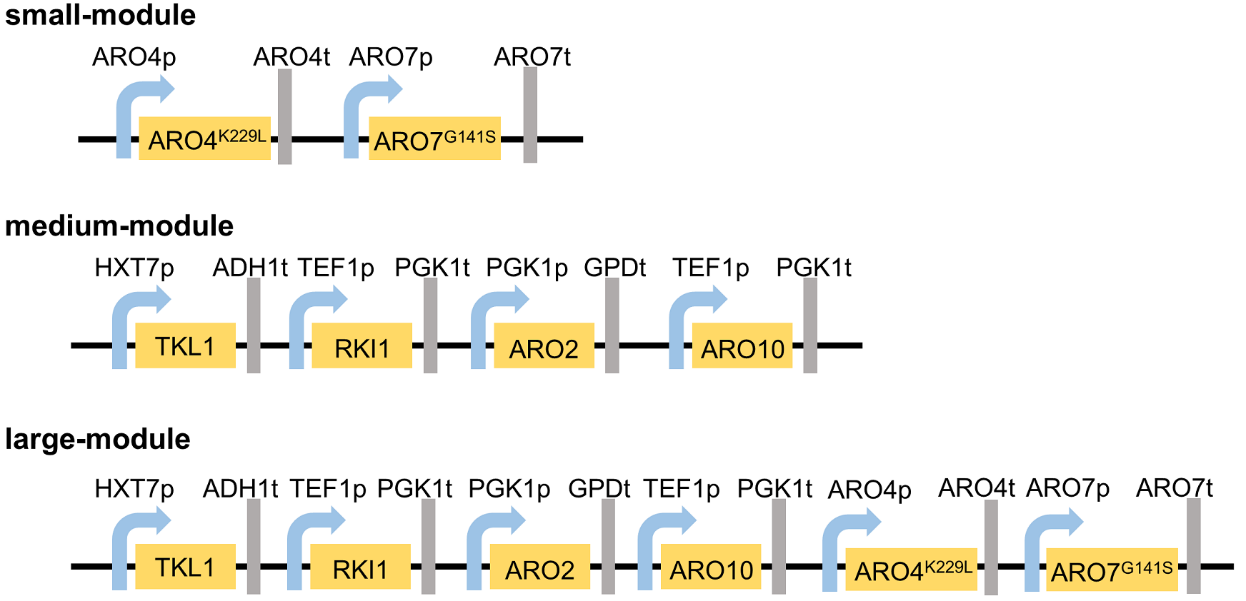


**Supplementary Figure 2.** Structures of modules with tyrosol pathway-related genes


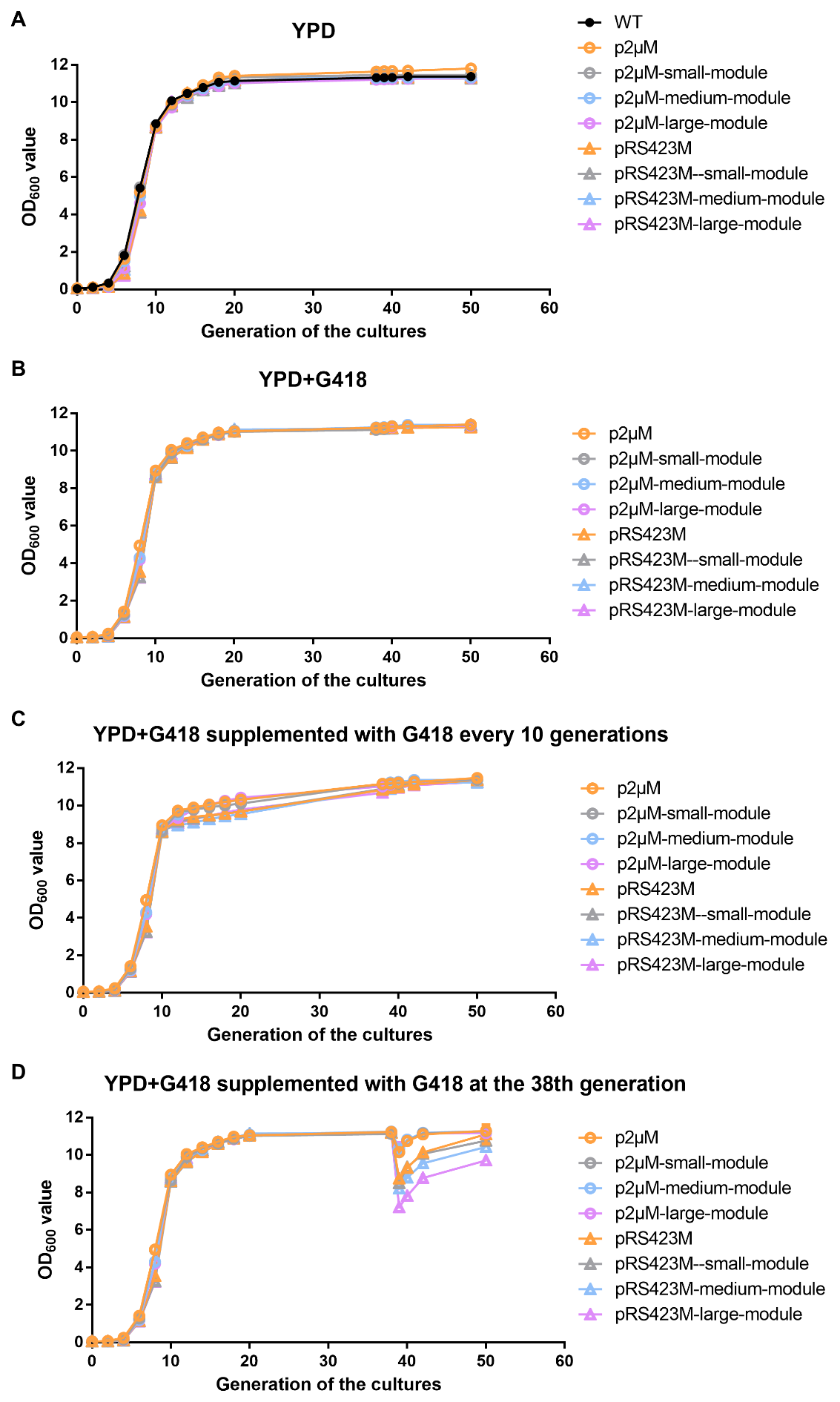


**Supplementary Figure 3.** Growth curves of strains under different culture conditions. **(A)** Cultures grown in YPD medium without G418. **(B)** Cultures grown in YPD+G418 medium. **(C)** Cultures grown in YPD+G418 medium supplemented with G418 every 10 generations. **(D)** Cultures grown in YPD+G418 medium supplemented with G418 at the 38th generation.

## 2.2 Supplementary Tables

**Supplementary Table 1.** Comparison of plasmids characteristics in YPD medium

| **Plasmids** | **Inserted genes** | **Size of modules**  **(bp)** | **Plasmid loss rate** **of the 10th generation**  **(%)** | **Plasmid loss rate** **of the 20th generation**  **(%)** | **Plasmid loss rate** **of the 40th generation**  **(%)** | **Plasmid loss rate** **of the 50th generation**  **(%)** |
| --- | --- | --- | --- | --- | --- | --- |
| p2μM | *KanMX* | 0 | 36.3 ± 6.0 | 62.4 ± 3.3 | 72.5 ± 7.9 | 85.7 ± 1.4 |
| p2μM-  small-module | *KanMX, ARO4^K229L^ , ARO7^G141S^* | 3842 | 54.3 ± 8.5 | 87.9 ± 2.4 | 91.9 ± 1.0 | 96.4 ± 0.9 |
| p2μM-  medium-module | *KanMX*, *TKL1*, *RKI1*, *ARO10*, *ARO2* | 9821 | 71.4 ± 5.6 | 95.8 ± 1.3 | 96.9 ± 0.8 | 98.7 ± 0.3 |
| p2μM-  large-module | *KanMX*, *TKL1*, *RKI1*, *ARO10*, *ARO2, ARO4^K229L^ , ARO7^G141S^* | 13663 | 94.3 ± 1.2 | 99.7 ± 0.4 | 100.0 | 100.0 |
| pRS423M | *KanMX* | 0 | 90.4 ± 2.9 | 98.8 ± 0.9 | 99.3 ± 0.2 | 99.9 ± 0.2 |
| pRS423M-  small-module | *KanMX, ARO4^K229L^ , ARO7^G141S^* | 3842 | 98.1 ± 1.3 | 99.7 ± 0.4 | 99.8 ± 0.1 | 100.0 |
| pRS423M-  medium-module | *KanMX*, *TKL1*, *RKI1*, *ARO10*, *ARO2* | 9821 | 97.2 ± 1.1 | 100.0 | 100.0 | 100.0 |
| pRS423M-  large-module | *KanMX*, *TKL1*, *RKI1*, *ARO10*, *ARO2, ARO4^K229L^ , ARO7^G141S^* | 13663 | 99.6 ± 0.2 | 99.6 ± 0.3 | 100.0 | 100.0 |

**Supplementary Table 2.** Comparison of plasmids characteristics in YPD+G418 medium

| **Plasmids** | **Inserted genes** | **Size of modules**  **(bp)** | **Plasmid loss rate** **of the 10th generation**  **(%)** | **Plasmid loss rate** **of the 20th generation**  **(%)** | **Plasmid loss rate** **of the 40th generation**  **(%)** | **Plasmid loss rate** **of the 50th generation**  **(%)** |
| --- | --- | --- | --- | --- | --- | --- |
| p2μM | *KanMX* | 0 | 5.7 ± 1.3 | 7.2 ± 0.7 | 12.4 ± 0.8 | 27.1 ± 1.4 |
| p2μM-  small-module | *KanMX, ARO4^K229L^ , ARO7^G141S^* | 3842 | 9.5 ± 0.5 | 15.2 ± 1.3 | 28.2 ± 1.0 | 46.8 ± 0.8 |
| p2μM-  medium-module | *KanMX*, *TKL1*, *RKI1*, *ARO10*, *ARO2* | 9821 | 20.5 ± 2.1 | 34.2 ± 1.1 | 45.8 ± 1.6 | 49.3 ± 2.5 |
| p2μM-  large-module | *KanMX*, *TKL1*, *RKI1*, *ARO10*, *ARO2, ARO4^K229L^ , ARO7^G141S^* | 13663 | 46.8 ± 1.2 | 53.6 ± 1.2 | 57.0 ± 1.9 | 66.6 ± 1.3 |
| pRS423M | *KanMX* | 0 | 17.8 ± 1.1 | 31.4 ± 1.8 | 74.8 ± 0.9 | 85.1 ± 2.2 |
| pRS423M-  small-module | *KanMX, ARO4^K229L^ , ARO7^G141S^* | 3842 | 37.0 ± 0.4 | 46.2 ± 1.4 | 78.2 ± 1.5 | 88.5 ± 1.0 |
| pRS423M-  medium-module | *KanMX*, *TKL1*, *RKI1*, *ARO10*, *ARO2* | 9821 | 47.5 ± 2.7 | 57.3 ± 1.7 | 87.1 ± 1.8 | 94.9 ± 1.3 |
| pRS423M-  large-module | *KanMX*, *TKL1*, *RKI1*, *ARO10*, *ARO2, ARO4^K229L^ , ARO7^G141S^* | 13663 | 66.8 ± 2.6 | 79.0 ± 2.6 | 98.6 ± 1.2 | 99.4 ± 0.6 |

**Supplementary Table 3.** Comparison of plasmids characteristics in YPD+G418 medium supplemented with G418 every 10 generations

| **Plasmids** | **Inserted genes** | **Size of modules**  **(bp)** | **Plasmid loss rate** **of the 10th generation**  **(%)** | **Plasmid loss rate** **of the 20th generation**  **(%)** | **Plasmid loss rate** **of the 40th generation**  **(%)** | **Plasmid loss rate** **of the 50th generation**  **(%)** |
| --- | --- | --- | --- | --- | --- | --- |
| p2μM | *KanMX* | 0 | 5.7 ± 1.3 | 7.1 ± 1.3 | 12.9 ± 0.3 | 18.8 ± 0.6 |
| p2μM-  small-module | *KanMX, ARO4^K229L^ , ARO7^G141S^* | 3842 | 9.5 ± 0.5 | 11.8 ± 0.4 | 22.0 ± 0.4 | 27.1 ± 0.5 |
| p2μM-  medium-module | *KanMX*, *TKL1*, *RKI1*, *ARO10*, *ARO2* | 9821 | 20.5 ± 2.1 | 12.4 ± 0.7 | 25.2 ± 0.4 | 33.5 ± 0.9 |
| p2μM-  large-module | *KanMX*, *TKL1*, *RKI1*, *ARO10*, *ARO2, ARO4^K229L^ , ARO7^G141S^* | 13663 | 46.8 ± 1.2 | 21.2 ± 0.6 | 31.2 ± 0.2 | 38.2 ± 0.8 |
| pRS423M | *KanMX* | 0 | 17.8 ± 1.1 | 25.6 ± 0.8 | 45.4 ± 1.0 | 61.7 ± 1.1 |
| pRS423M-  small-module | *KanMX, ARO4^K229L^ , ARO7^G141S^* | 3842 | 37.0 ± 0.4 | 43.0 ± 0.2 | 56.9 ± 0.7 | 74.3 ± 1.1 |
| pRS423M-  medium-module | *KanMX*, *TKL1*, *RKI1*, *ARO10*, *ARO2* | 9821 | 47.5 ± 2.7 | 55.7 ± 1.6 | 65.1 ± 0.4 | 79.4 ± 0.4 |
| pRS423M-  large-module | *KanMX*, *TKL1*, *RKI1*, *ARO10*, *ARO2, ARO4^K229L^ , ARO7^G141S^* | 13663 | 66.8 ± 2.6 | 72.6 ± 2.0 | 82.2 ± 0.8 | 86.9 ± 0.1 |

**Supplementary Table 4.** Comparison of plasmids characteristics in YPD+G418 medium supplemented with G418 at the 38th generation

| **Plasmids** | **Inserted genes** | **Size of modules**  **(bp)** | **Plasmid loss rate** **of the 10th generation**  **(%)** | **Plasmid loss rate** **of the 20th generation**  **(%)** | **Plasmid loss rate** **of the 40th generation**  **(%)** | **Plasmid loss rate** **of the 50th generation**  **(%)** |
| --- | --- | --- | --- | --- | --- | --- |
| p2μM | *KanMX* | 0 | 5.7 ± 1.3 | 7.2 ± 0.7 | 2.2 ± 0.5 | 4.0 ± 0.1 |
| p2μM-  small-module | *KanMX, ARO4^K229L^ , ARO7^G141S^* | 3842 | 9.5 ± 0.5 | 15.2 ± 1.3 | 3.5 ± 0.2 | 10.3 ± 0.8 |
| p2μM-  medium-module | *KanMX*, *TKL1*, *RKI1*, *ARO10*, *ARO2* | 9821 | 20.5 ± 2.1 | 34.2 ± 1.1 | 6.9 ± 0.8 | 20.3 ± 1.5 |
| p2μM-  large-module | *KanMX*, *TKL1*, *RKI1*, *ARO10*, *ARO2, ARO4^K229L^ , ARO7^G141S^* | 13663 | 46.8 ± 1.2 | 53.6 ± 1.2 | 12.2 ± 0.5 | 33.2 ± 1.0 |
| pRS423M | *KanMX* | 0 | 17.8 ± 1.1 | 31.4 ± 1.8 | 29.4 ± 0.7 | 55.2 ± 2.5 |
| pRS423M-  small-module | *KanMX, ARO4^K229L^ , ARO7^G141S^* | 3842 | 37.0 ± 0.4 | 46.2 ± 1.4 | 43.9 ± 0.4 | 68.7 ± 0.5 |
| pRS423M-  medium-module | *KanMX*, *TKL1*, *RKI1*, *ARO10*, *ARO2* | 9821 | 47.5 ± 2.7 | 57.3 ± 1.7 | 55.0 ± 0.4 | 81.0 ± 1.0 |
| pRS423M-  large-module | *KanMX*, *TKL1*, *RKI1*, *ARO10*, *ARO2, ARO4^K229L^ , ARO7^G141S^* | 13663 | 66.8 ± 2.6 | 79.0 ± 2.6 | 75.4 ± 0.5 | 90.3 ± 0.4 |

**Supplementary Table 5.** Strains used in this study

| **Strains** | **Characteristics** |
| --- | --- |
| **DH5α** | *F̶ ϕ80dlacZΔM15 endAI recAI hsd RI7(r_K_^-^m_K_^+^) supE44 thi-1 relAI*  *Δ (lacZYA-argF) U169 gyrA96 deoR.* |
| **DH10B** | *F-mcrA (mrr-hsdRMS-mcrBC) φ80lacZ.M15 lacX74 recA1 endA1 ara-*  *D139 (ara, leu)7697 galU galK λ- rpsL nupG /pMON14272/pMON7124* |
| **BY4741α** | *MATa his3Δ1 leu2Δ0 met15Δ0 ura3Δ0* |
| **CEN.PK2**-**1C** | *MATa ura3-52 his3-Δ1 leu2-3_112 trp1-289, MAL2-8c SUC2* |
| CEN.PK2-1C  p2μM | CEN.PK2-1C, p2μ-*TEF1*p*-KanMX-TEF2*t |
| CEN.PK2-1C  p2μM-small-module | CEN.PK2-1C, p2μ-*KanMX-ARO4^K229L^ ARO7^G141S^* |
| CEN.PK2-1C  p2μM-medium-module | CEN.PK2-1C, p2μ*-KanMX-TKL1-RKI1-ARO10-ARO2* |
| CEN.PK2-1C  p2μM-large-module | CEN.PK2-1C, p2μ-*KanMX-TKL1-RKI1-ARO10-ARO2-ARO4^K229L^-ARO7^G141S^* |
| CEN.PK2-1C pRS423M | CEN.PK2-1C, pRS423*-TEF1*p*-KanMX-TEF2*t |
| CEN.PK2-1C pRS423M-small-module | CEN.PK2-1C, pRS423-*KanMX-ARO4^K229L^-ARO7^G141S^* |
| CEN.PK2-1C pRS423M-medium-module | CEN.PK2-1C, pRS423-*KanMX-TKL1-RKI1-ARO10-ARO2* |
| CEN.PK2-1C pRS423M- large-module | CEN.PK2-1C, pRS423-*KanMX-TKL1-RKI1-ARO10-ARO2-ARO4^K229L^ -ARO7^G141S^* |

Bold strains were all previously existing in this laboratory, those not in bold were strains constructed by the authors.

**Supplementary Table 6.** Plasmids used in this study

| **Plasmid name** | **Relevant Features** |
| --- | --- |
| p2μ | Non-selection marker |
| p2μM | p2μ-*TEF1*p-*KanMX*-*TEF2*t |
| p2μM-small-module | p2μ-*TEF1*p*-KanMX-TEF2*t-*ARO4*p*-ARO4^K229L^-ARO4*t-*ARO7*p-*ARO7^G141S^-ARO7*t |
| p2μM-medium-module | p2μ-*TEF1*p-*KanMX*-*TEF2*t-*HXT7*p-*TKL1*1-*ADH1*t-*TEF1*p-*RKI1*-*PGK1*t-*PGK1*p-*ARO2*-*GPD*t-*TEF1*p-*ARO10*-*PGK1*t |
| p2μM-large-module | p2μ-*TEF1*p-*KanMX*-*TEF2*t-*HXT7*p-*TKL1*1-*ADH1*t-*TEF1*p-*RKI1*-*PGK1*t-*PGK1*p-*ARO2*-*GPD*t-*TEF1*p-*ARO10*-*PGK1*t-*ARO4*p-*ARO4^K229L^*-*ARO4*t-*ARO7*p-*ARO7^G141S^*-*ARO7*t |
| pRS423 | *His3* marker, 2μ origin |
| pRS423M | pRS423-*TEF1*p-*KanMX*-*TEF2*t |
| pRS423M-small-module | pRS423-*TEF1*p-*KanMX*-*TEF2*t-*ARO4*p-*ARO4^K229L^*-*ARO4*t-*ARO7*p-*ARO7^G141S^*-*ARO7*t |
| pRS423M-medium-module | pRS423-*TEF1*p-*KanMX*-*TEF2*t-*HXT7*p-*TKL1*1-*ADH1*t-*TEF1*p-*RKI1*-*PGK1*t-*PGK1*p-*ARO2*-*GPD*t-*TEF1*p-*ARO10*-*PGK1*t |
| pRS423M-large-module | pRS423-*TEF1*p-*KanMX*-*TEF2*t-*HXT7*p-*TKL1*1-*ADH1*t-*TEF1*p-*RKI1*-*PGK1*t-*PGK1*p-*ARO2*-*GPD*t-*TEF1*p-*ARO10*-*PGK1*t-*ARO4*p-*ARO4^K229L^*-*ARO4*t-*ARO7*p-*ARO7^G141S^*-*ARO7*t |

**Supplementary Table 7.** Primers used in this study

| Name | Sequence (5’->3’) |
| --- | --- |
| F1-2μ (*REP2*+30a) | cagattcttggctgtttcaatgtcgtccat |
| R1-2μ (*REF1*p+41a) | gggcggagcctatggaaagctggggtgattgctcgatttcg |
| F2-2μ (*RAF1*p+48a) | cgcggatccgtttaaacctcgagcgaaatcgagcaatcaccccagctg |
| R2-2μ (*REP2*+28a) | ggacgacattgaaacagccaagaatctg |
| F3-pBR322+26a | cagctttccataggctccgcccccct |
| R3-pBR322+30a | ttgagatcctttttttctgcgcgtaatctg |
| F4-*TEF1*p+51a | cagattacgcgcagaaaaaaaggatctcaagacatggaggcccagaatacc |
| R4-*TEF2*t+54a | ctcgaggtttaaacggatccgcggccgccagtatagcgaccagcattcacatac |
| F10-*HXT7*p+52a | gtgaatgctggtcgctatactggcggccgcacttctcgtaggaacaatttcg |
| R16-*PGK1*t+59a | gctcgatttcgctcgaggtttaaacggatccaacgcagaattttcgagttattaaactt |
| F13-*ARO4*p+55a | gtgaatgctggtcgctatactggcggccgctagtcaaacaaaaaagggaaattct |
| R13-*ARO7*t+58a | cgctcgaggtttaaacggatcccgtttaagaaaatagtacaatataatgtataacttg |
| F11-CCW12p+51a | gtgaatgctggtcgctatactggcggccgcggatacttcatgctatttatagacg |
| R11-*GPD*t+45a | cgctcgaggtttaaacggatccggaatctgtgtatattactgcatctag |
| F12-*TDH3*p+56a | gtgaatgctggtcgctatactggcggccgctcattatcaatactgccatttcaaag |
| R12-*FBA1*t+57a | cgctcgaggtttaaacggatccaaagatgagctaggcttttgtaaaaatatcttacg |
| F15-*CCW12*p+39a | cgaaaattctgcgttggatacttcatgctatttatagac |
| R15-*PGK1*t+25a | gcatgaagtatccaacgcagaattttcg |
| F16-*HXT7*p+58a | tcgtatgtgaatgctggtcgctatactggcggccgcacttctcgtaggaacaatttcg |
| F16-*TDH3*p+50a | gatgcagtaatatacacagattcctcattatcaatactgccatttcaaag |
| R15-*ARO7*t+24a | atttcgctcgaggtttaaacggat |
| F18-*ARO4*p+56a | gatgcagtaatatacacagattcctagtcaaacaaaaaagggaaattctatttgtc |
| F15-*ARO4*p+52a | caaaagcctagctcatcttttagtcaaacaaaaaagggaaattctatttgtc |
| F9-seqin*TEF2*t+20a | tcgacatcatctgcccagat |
| R9-seqin*RAF1*p+20a | gccttaatcccttctcgaag |

# 3 Growth of the cultures

According to **Supplementary Figure 3**, in YPD medium, YPD+G418 medium and YPD+G418 medium supplemented with G418 every 10 generations, the growth of the cultures followed the general law of the microbial growth cycle. Supplementing antibiotics every 10 generations did not significantly inhibit the growth of the cultures. The OD_600_ values of the cultures in stable period (from the 10th to the 50th generation) reached about 11.50. After the 38th generation cultures were supplemented with G418, the OD_600_ values of all cultures decreased obviously, and that of the cultures containing pRS423M-based plasmids decreased most significantly (from 11.19 ± 0.03 at the 38th generation to 7.22 ± 0.03 at the 39th generation), which indicating that more strains without resistance genes were killed by the supplemented antibiotics. Changes in plasmid loss rates and OD_600_ values also indicated that the original antibiotic lost its screening effect.

# 4 The Complete Sequences of Plasmids Constructed in This Study

## 4.1 Sequence of plasmid p2μM

5’-gaattctgaaccagtcctaaaacgagtaaataggaccggcaattcttcaagcaataaacaggaataccaattattaaaa

gataacttagtcagatcgtacaataaagctttgaagaaaaatgcgccttattcaatctttgctataaaaaatggcccaaaatctcacattggaagacatttgatgacctcatttctttcaatgaagggcctaacggagttgactaatgttgtgggaaattggagcgataagcgtgcttctgccgtggccaggacaacgtatactcatcagataacagcaatacctgatcactacttcgcactagtttctcggtactatgcatatgatccaatatcaaaggaaatgatagcattgaaggatgagactaatccaattgaggagtggcagcatatagaacagctaaagggtagtgctgaaggaagcatacgataccccgcatggaatgggataatatcacaggaggtactagactacctttcatcctacataaatagacgcatataagtacgcatttaagcataaacacgcactatgccgttcttctcatgtatatatatatacaggcaacacgcagatataggtgcgacgtgaacagtgagctgtatgtgcgcagctcgcgttgcattttcggaagcgctcgttttcggaaacgctttgaagttcctattccgaagttcctattctctagaaagtataggaacttcagagcgcttttgaaaaccaaaagcgctctgaagacgcactttcaaaaaaccaaaaacgcaccggactgtaacgagctactaaaatattgcgaataccgcttccacaaacattgctcaaaagtatctctttgctatatatctctgtgctatatccctatataacctacccatccacctttcgctccttgaacttgcatctaaactcgacctctacatcaacaggcttccaatgctcttcaaattttactgtcaagtagacccatacggctgtaatatgctgctcttcataatgtaagcttatctttatcgaatcgtgtgaaaaactactaccgcgataaacctttacggttccctgagattgaattagttcctttagtatatgatacaagacacttttgaactttgtacgacgaattttgaggttcgccatcctctggctatttccaattatcctgtcggctattatctccgcctcagtttgatcttccgcttcagactgccatttttcacataatgaatctatttcaccccacaatccttcatccgcctccgcatcttgttccgttaaactattgacttcatgttgtacattgtttagttcacgagaagggtcctcttcaggcggtagctcctgatctcctatatgacctttatcctgttctctttccacaaacttagaaatgtattcatgaattatggagcacctaataacattcttcaaggcggagaagtttgggccagatgcccaatatgcttgacatgaaaacgtgagaatgaatttagtattattgtgatattctgaggcaattttattataatctcgaagataagagaagaatgcagtgacctttgtattgacaaatggagattccatgtatctaaaaaatacgcctttaggccttctgataccctttcccctgcggtttagcgtgccttttacattaatatctaaaccctctccgatggtggcctttaactgactaataaatgcaaccgatataaactgtgataattctgggtgatttatgattcgatcgacaattgtattgtacactagtgcaggatcaggccaatccagttctttttcaattaccggtgtgtcgtctgtattcagtacatgtccaacaaatgcaaatgctaacgttttgtatttcttataattgtcaggaactggaaaagtcccccttgtcgtctcgattacacacctactttcatcgtacaccataggttggaagtgctgcataatacattgcttaatacaagcaagcagtctctcgccattcatatttcagttattttccattacagctgatgtcattgtatatcagcgctgtaaaaatctatctgttacagaaggttttcgcggtttttataaacaaaactttcgttacgaaatcgagcaatcaccccagctttccataggctccgcccccctgacgagcatcacaaaaatcgacgctcaagtcagaggtggcgaaacccgacaggactataaagataccaggcgtttccccctggaagctccctcgtgcgctctcctgttccgaccctgccgcttaccggatacctgtccgcctttctcccttcgggaagcgtggcgctttctcatagctcacgctgtaggtatctcagttcggtgtaggtcgttcgctccaagctgggctgtgtgcacgaaccccccgttcagcccgaccgctgcgccttatccggtaactatcgtcttgagtccaacccggtaagacacgacttatcgccactggcagcagccactggtaacaggattagcagagcgaggtatgtaggcggtgctacagagttcttgaagtggtggcctaactacggctacactagaaggacagtatttggtatctgcgctctgctgaagccagttaccttcggaaaaagagttggtagctcttgatccggcaaacaaaccaccgctggtagcggtggtttttttgtttgcaagcagcagattacgcgcagaaaaaaaggatctcaagacatggaggcccagaataccctccttgacagtcttgacgtgcgcagctcaggggcatgatgtgactgtcgcccgtacatttagcccatacatccccatgtataatcatttgcatccatacattttgatggccgcacggcgcgaagcaaaaattacggctcctcgctgcagacctgcgagcagggaaacgctcccctcacagacgcgttgaattgtccccacgccgcgcccctgtagagaaatataaaaggttaggatttgccactgaggttcttctttcatatacttccttttaaaatcttgctaggatacagttctcacatcacatccgaacataaacaaccatgggtaaggaaaagactcacgtttcgaggccgcgattaaattccaacatggatgctgatttatatgggtataaatgggctcgcgataatgtcgggcaatcaggtgcgacaatctatcgattgtatgggaagcccgatgcgccagagttgtttctgaaacatggcaaaggtagcgttgccaatgatgttacagatgagatggtcagactaaactggctgacggaatttatgcctcttccgaccatcaagcattttatccgtactcctgatgatgcatggttactcaccactgcgatccccggcaaaacagcattccaggtattagaagaatatcctgattcaggtgaaaatattgttgatgcgctggcagtgttcctgcgccggttgcattcgattcctgtttgtaattgtccttttaacagcgatcgcgtatttcgtctcgctcaggcgcaatcacgaatgaataacggtttggttgatgcgagtgattttgatgacgagcgtaatggctggcctgttgaacaagtctggaaagaaatgcataagcttttgccattctcaccggattcagtcgtcactcatggtgatttctcacttgataaccttatttttgacgaggggaaattaataggttgtattgatgttggacgagtcggaatcgcagaccgataccaggatcttgccatcctatggaactgcctcggtgagttttctccttcattacagaaacggctttttcaaaaatatggtattgataatcctgatatgaataaattgcagtttcatttgatgctcgatgagtttttctaatcagtactgacaataaaaagattcttgttttcaagaacttgtcatttgtatagtttttttatattgtagttgttctattttaatcaaatgttagcgtgatttatattttttttcgcctcgacatcatctgcccagatgcgaagttaagtgcgcagaaagtaatatcatgcgtcaatcgtatgtgaatgctggtcgctatactggcggccgcggatccgtttaaacctcgagcgaaatcgagcaatcaccccagctgcgtatttggaaattcgggaaaaagtagagcaacgcgagttgcattttttacaccataatgcatgattaacttcgagaagggattaaggctaatttcactagtatgtttcaaaaacctcaatctgtccattgaatgccttataaaacagctatagattgcatagaagagttagctactcaatgctttttgtcaaagcttactgatgatgatgtgtctactttcaggcgggtctgtagtaaggagaatgacattataaagctggcacttagaattccacggactatagactatactagtatactccgtctactgtacgatacacttccgctcaggtccttgtcctttaacgaggccttaccactcttttgttactctattgatccagctcagcaaaggcagtgtgatctaagattctatcttcgcgatgtagtaaaactagctagaccgagaaagagactagaaatgcaaaaggcacttctacaatggctgccatcattattatccgatgtgacgctgcagcttctcaatgatattcgaatacgctttgaggagatacagcctaatatccgacaaactgttttacagatttacgatcgtacttgttacccatcattgaattttgaacatccgaacctgggagttttccctgaaacagatagtatatttgaacctgtataataatatatagtctagcgctttacggaagacaatgtatgtatttcggttcctggagaaactattgcatctattgcataggtaatcttgcacgtcgcatccccggttcattttctgcgtttccatcttgcacttcaatagcatatctttgttaacgaagcatctgtgcttcattttgtagaacaaaaatgcaacgcgagagcgctaatttttcaaacaaagaatctgagctgcatttttacagaacagaaatgcaacgcgaaagcgctattttaccaacgaagaatctgtgcttcatttttgtaaaacaaaaatgcaacgcgagagcgctaatttttcaaacaaagaatctgagctgcatttttacagaacagaaatgcaacgcgagagcgctattttaccaacaaagaatctatacttcttttttgttctacaaaaatgcatcccgagagcgctatttttctaacaaagcatcttagattactttttttctcctttgtgcgctctataatgcagtctcttgataactttttgcactgtaggtccgttaaggttagaagaaggctactttggtgtctattttctcttccataaaaaaagcctgactccacttcccgcgtttactgattactagcgaagctgcgggtgcattttttcaagataaaggcatccccgattatattctataccgatgtggattgcgcatactttgtgaacagaaagtgatagcgttgatgattcttcattggtcagaaaattatgaacggtttcttctattttgtctctatatactacgtataggaaatgtttacattttcgtattgttttcgattcactctatgaatagttcttactacaatttttttgtctaaagagtaatactagagataaacataaaaaatgtagaggtcgagtttagatgcaagttcaaggagcgaaaggtggatgggtaggttatatagggatatagcacagagatatatagcaaagagatacttttgagcaatgtttgtggaagcggtattcgcaatattttagtagctcgttacagtccggtgcgtttttggttttttgaaagtgcgtcttcagagcgcttttggttttcaaaagcgctctgaagttcctatactttctagagaataggaacttcggaataggaacttcaaagcgtttccgaaaacgagcgcttccgaaaatgcaacgcgagctgcgcacatacagctcactgttcacgtcgcacctatatctgcgtgttgcctgtatatatatatacatgagaagaacggcatagtgcgtgtttatgcttaaatgcgtacttatatgcgtctatttatgtaggatgaaaggtagtctagtacctcctgtgatattatcccattccatgcggggtatcgtatgcttccttcagcactaccctttagctgttctatatgctgccactcctcaattggattagtctcatccttcaatgctatcatttcctttgatattggatcataccctagaagtattacgtgattttctgccccttaccctcgttgctactctcctttttttcgtgggaaccgctttagggccctcagtgatggtgttttgtaatttatatgctcctcttgcatttgtgtctctacttcttgttcgcctggagggaacttcttcatttgtattagcatggttcacttcagtccttccttccaactcactctttttttgctgtaaacgattctctgccgccagttcattgaaactattgaatatatcctttagagattccgggatgaataaatcacctattaaagcagcttgacgatctggtggaactaaagtaagcaattgggtaacgacgcttacgagcttcataacatcttcttccgttggagctggtgggactaataactgtgtacaatccatttttctcatgagcatttcggtagctctcttcttgtctttctcgggcaatcttcctattattatagcaatagatttgtatagttgctttctattgtctaacagcttgttattctgtagcatcaaatctatggcagcctgacttgcttcttgtgaagagagcataccatttccaatcgaatcaaacctttccttaaccatcttcgcagcaggcaaaattacctcagcactggagtcagaagatacgctggaatcttctgcgctagaatcaagaccatacggcctaccggttgtgagagattccatgggccttatgacatatcctggaaagagtagctcatcagacttacgtttactctctatatcaatatctacatcaggagcaatcatttcaataaacagccgacatacatcccagacgctataagctgtacgtgcttttaccgtcagattcttggctgtttcaatgtcgtccattttggttttcttttaccagtattgttcgtttgataatgtattcttgcttattacattataaaatctgtgcagatcacatgtcaaaacaactttttatcacaagatagtaccgcaaaacgaacctgcgggccgtctaaaaattaaggaaaagcagcaaaggtgcatttttaaaatatgaaatgaagataccgcagtaccaattattttcgcagtacaaataatgcgcggccggtgcatttttcgaaagaacgcgagacaaacaggacaattaaagttagtttttcgagttagcgtgtttgaatactgcaagatacaagataaatagagtagttgaaactagatatcaattgcacacaagatcggcgctaagcatgccacaatttggtatattatgtaaaacaccacctaaggtgcttgttcgtcagtttgtggaaaggtttgaaagaccttcaggtgagaaaatagcattatgtgctgctgaactaacctatttatgttggatgattacacataacggaacagcaatcaagagagccacattcatgagctataatactatcataagcaattcgctgagtttcgatattgtcaataaatcactccagtttaaatacaagacgcaaaaagcaacaattctggaagcctcattaaagaaattgattcctgcttgggaatttacaattattccttactatggacaaaaacatcaatctgatatcactgatattgtaagtagtttgcaattacagttcgaatcatcggaagaagcagataagggaaatagccacagtaaaaaaatgcttaaagcacttctaagtgagggtgaaagcatctgggagatcactgagaaaatactaaattcgtttgagtatacttcgagatttacaaaaacaaaaactttataccaattcctcttcctagctactttcatcaattgtggaagattcagcgatattaagaacgttgatccgaaatcatttaaattagtccaaaataagtatctgggagtaataatccagtgtttagtgacagagacaaagacaagcgttagta

ggcacatatacttctttagcgcaaggggtaggatcgatccacttgtatatttggatgaatttttgag-3’

## 4.2 Sequence of plasmid p423M

5’-gacgaaagggcctcgtgatacgcctatttttataggttaatgtcatgataataatggtttcttagatgatccaatatcaaag

gaaatgatagcattgaaggatgagactaatccaattgaggagtggcagcatatagaacagctaaagggtagtgctgaaggaagcatacgataccccgcatggaatgggataatatcacaggaggtactagactacctttcatcctacataaatagacgcatataagtacgcatttaagcataaacacgcactatgccgttcttctcatgtatatatatatacaggcaacacgcagatataggtgcgacgtgaacagtgagctgtatgtgcgcagctcgcgttgcattttcggaagcgctcgttttcggaaacgctttgaagttcctattccgaagttcctattctctagaaagtataggaacttcagagcgcttttgaaaaccaaaagcgctctgaagacgcactttcaaaaaaccaaaaacgcaccggactgtaacgagctactaaaatattgcgaataccgcttccacaaacattgctcaaaagtatctctttgctatatatctctgtgctatatccctatataacctacccatccacctttcgctccttgaacttgcatctaaactcgacctctacattttttatgtttatctctagtattactctttagacaaaaaaattgtagtaagaactattcatagagtgaatcgaaaacaatacgaaaatgtaaacatttcctatacgtagtatatagagacaaaatagaagaaaccgttcataattttctgaccaatgaagaatcatcaacgctatcactttctgttcacaaagtatgcgcaatccacatcggtatagaatataatcggggatgcctttatcttgaaaaaatgcacccgcagcttcgctagtaatcagtaaacgcgggaagtggagtcaggctttttttatggaagagaaaatagacaccaaagtagccttcttctaaccttaacggacctacagtgcaaaaagttatcaagagactgcattatagagcgcacaaaggagaaaaaaagtaatctaagatgctttgttagaaaaatagcgctctcgggatgcatttttgtagaacaaaaaagaagtatagattctttgttggtaaaatagcgctctcgcgttgcatttctgttctgtaaaaatgcagctcagattctttgtttgaaaaattagcgctctcgcgttgcatttttgttttacaaaaatgaagcacagattcttcgttggtaaaatagcgctttcgcgttgcatttctgttctgtaaaaatgcagctcagattctttgtttgaaaaattagcgctctcgcgttgcatttttgttctacaaaatgaagcacagatgcttcgttcaggtggcacttttcggggaaatgtgcgcggaacccctatttgtttatttttctaaatacattcaaatatgtatccgctcatgagacaataaccctgataaatgcttcaataatattgaaaaaggaagagtatgagtattcaacatttccgtgtcgcccttattcccttttttgcggcattttgccttcctgtttttgctcacccagaaacgctggtgaaagtaaaagatgctgaagatcagttgggtgcacgagtgggttacatcgaactggatctcaacagcggtaagatccttgagagttttcgccccgaagaacgttttccaatgatgagcacttttaaagttctgctatgtggcgcggtattatcccgtattgacgccgggcaagagcaactcggtcgccgcatacactattctcagaatgacttggttgagtactcaccagtcacagaaaagcatcttacggatggcatgacagtaagagaattatgcagtgctgccataaccatgagtgataacactgcggccaacttacttctgacaacgatcggaggaccgaaggagctaaccgcttttttgcacaacatgggggatcatgtaactcgccttgatcgttgggaaccggagctgaatgaagccataccaaacgacgagcgtgacaccacgatgcctgtagcaatggcaacaacgttgcgcaaactattaactggcgaactacttactctagcttcccggcaacaattaatagactggatggaggcggataaagttgcaggaccacttctgcgctcggcccttccggctggctggtttattgctgataaatctggagccggtgagcgtgggtctcgcggtatcattgcagcactggggccagatggtaagccctcccgtatcgtagttatctacacgacggggagtcaggcaactatggatgaacgaaatagacagatcgctgagataggtgcctcactgattaagcattggtaactgtcagaccaagtttactcatatatactttagattgatttaaaacttcatttttaatttaaaaggatctaggtgaagatcctttttgataatctcatgaccaaaatcccttaacgtgagttttcgttccactgagcgtcagaccccgtagaaaagatcaaaggatcttcttgagatcctttttttctgcgcgtaatctgctgcttgcaaacaaaaaaaccaccgctaccagcggtggtttgtttgccggatcaagagctaccaactctttttccgaaggtaactggcttcagcagagcgcagataccaaatactgtccttctagtgtagccgtagttaggccaccacttcaagaactctgtagcaccgcctacatacctcgctctgctaatcctgttaccagtggctgctgccagtggcgataagtcgtgtcttaccgggttggactcaagacgatagttaccggataaggcgcagcggtcgggctgaacggggggttcgtgcacacagcccagcttggagcgaacgacctacaccgaactgagatacctacagcgtgagctatgagaaagcgccacgcttcccgaagggagaaaggcggacaggtatccggtaagcggcagggtcggaacaggagagcgcacgagggagcttccagggggaaacgcctggtatctttatagtcctgtcgggtttcgccacctctgacttgagcgtcgatttttgtgatgctcgtcaggggggcggagcctatggaaaaacgccagcaacgcggcctttttacggttcctggccttttgctggccttttgctcacatgttctttcctgcgttatcccctgattctgtggataaccgtattaccgcctttgagtgagctgataccgctcgccgcagccgaacgaccgagcgcagcgagtcagtgagcgaggaagcggaagagcgcccaatacgcaaaccgcctctccccgcgcgttggccgattcattaatgcagctggcacgacaggtttcccgactggaaagcgggcagtgagcgcaacgcaattaatgtgagttacctcactcattaggcaccccaggctttacactttatgcttccggctcctatgttgtgtggaattgtgagcggataacaatttcacacaggaaacagctatgaccatgattacgccaagcgcgcaattaaccctcactaaagggaacaaaagctggagctccaccgcggtggcggccgctctagaactagtgacatggaggcccagaataccctccttgacagtcttgacgtgcgcagctcaggggcatgatgtgactgtcgcccgtacatttagcccatacatccccatgtataatcatttgcatccatacattttgatggccgcacggcgcgaagcaaaaattacggctcctcgctgcagacctgcgagcagggaaacgctcccctcacagacgcgttgaattgtccccacgccgcgcccctgtagagaaatataaaaggttaggatttgccactgaggttcttctttcatatacttccttttaaaatcttgctaggatacagttctcacatcacatccgaacataaacaaccatgggtaaggaaaagactcacgtttcgaggccgcgattaaattccaacatggatgctgatttatatgggtataaatgggctcgcgataatgtcgggcaatcaggtgcgacaatctatcgattgtatgggaagcccgatgcgccagagttgtttctgaaacatggcaaaggtagcgttgccaatgatgttacagatgagatggtcagactaaactggctgacggaatttatgcctcttccgaccatcaagcattttatccgtactcctgatgatgcatggttactcaccactgcgatccccggcaaaacagcattccaggtattagaagaatatcctgattcaggtgaaaatattgttgatgcgctggcagtgttcctgcgccggttgcattcgattcctgtttgtaattgtccttttaacagcgatcgcgtatttcgtctcgctcaggcgcaatcacgaatgaataacggtttggttgatgcgagtgattttgatgacgagcgtaatggctggcctgttgaacaagtctggaaagaaatgcataagcttttgccattctcaccggattcagtcgtcactcatggtgatttctcacttgataaccttatttttgacgaggggaaattaataggttgtattgatgttggacgagtcggaatcgcagaccgataccaggatcttgccatcctatggaactgcctcggtgagttttctccttcattacagaaacggctttttcaaaaatatggtattgataatcctgatatgaataaattgcagtttcatttgatgctcgatgagtttttctaatcagtactgacaataaaaagattcttgttttcaagaacttgtcatttgtatagtttttttatattgtagttgttctattttaatcaaatgttagcgtgatttatattttttttcgcctcgacatcatctgcccagatgcgaagttaagtgcgcagaaagtaatatcatgcgtcaatcgtatgtgaatgctggtcgctatactggatatcaagcttatcgataccgtcgacctcgagggggggcccggtacccaattcgccctatagtgagtcgtattacgcgcgctcactggccgtcgttttacaacgtcgtgactgggaaaaccctggcgttacccaacttaatcgccttgcagcacatccccctttcgccagctggcgtaatagcgaagaggcccgcaccgatcgcccttcccaacagttgcgcagcctgaatggcgaatggcgcgacgcgccctgtagcggcgcattaagcgcggcgggtgtggtggttacgcgcagcgtgaccgctacacttgccagcgccctagcgcccgctcctttcgctttcttcccttcctttctcgccacgttcgccggctttccccgtcaagctctaaatcgggggctccctttagggttccgatttagtgctttacggcacctcgaccccaaaaaacttgattagggtgatggttcacgtagtgggccatcgccctgatagacggtttttcgccctttgacgttggagtccacgttctttaatagtggactcttgttccaaactggaacaacactcaaccctatctcggtctattcttttgatttataagggattttgccgatttcggcctattggttaaaaaatgagctgatttaacaaaaatttaacgcgaattttaacaaaatattaacgtttacaatttcctgatgcggtattttctccttacgcatctgtgcggtatttcacaccgcatagatccgtcgagttcaagagaaaaaaaaagaaaaagcaaaaagaaaaaaggaaagcgcgcctcgttcagaatgacacgtatagaatgatgcattaccttgtcatcttcagtatcatactgttcgtatacatacttactgacattcataggtatacatatatacacatgtatatatatcgtatgctgcagctttaaataatcggtgtcactacataagaacacctttggtggagggaacatcgttggtaccattgggcgaggtggcttctcttatggcaaccgcaagagccttgaacgcactctcactacggtgatgatcattcttgcctcgcagacaatcaacgtggagggtaattctgctagcctctgcaaagctttcaagaaaatgcgggatcatctcgcaagagagatctcctactttctccctttgcaaaccaagttcgacaactgcgtacggcctgttcgaaagatctaccaccgctctggaaagtgcctcatccaaaggcgcaaatcctgatccaaacctttttactccacgcgccagtagggcctctttaaaagcttgaccgagagcaatcccgcagtcttcagtggtgtgatggtcgtctatgtgtaagtcaccaatgcactcaacgattagcgaccagccggaatgcttggccagagcatgtatcatatggtccagaaaccctatacctgtgtggacgttaatcacttgcgattgtgtggcctgttctgctactgcttctgcctctttttctgggaagatcgagtgctctatcgctaggggaccaccctttaaagagatcgcaatctgaatcttggtttcatttgtaatacgctttactagggctttctgctctgtcatctttgccttcgtttatcttgcctgctcattttttagtatattcttcgaagaaatcacattactttatataatgtataattcattatgtgataatgccaatcgctaagaaaaaaaaagagtcatccgctaggggaaaaaaaaaaatgaaaatcattaccgaggcataaaaaaatatagagtgtactagaggaggccaagagtaatagaaaaagaaaattgcgggaaaggactgtgttatgacttccctgactaatgccgtgttcaaacgatacctggcagtgactcctagcgctcaccaagctcttaaaacgggaatttatggtgcactctcagtacaatctgctctgatgccgcatagttaagccagccccgacacccgccaacacccgctgacgcgccctgacgggcttgtctgctcccggcatccgcttacagacaagctgtgaccgtctccgggagctgcatgtgtcagaggttttcacc

gtcatcaccgaaacgcgcga-3’

## 4.3 Sequence of the small-module

5’-tagtcaaacaaaaaagggaaattctatttgtctgattttgttgcaagataacaatactggcaaacatgggcttgagtgctt

aggaagaaatcttgaggtagcagtaacttctttttattaaacagtatgcagccgcccaaaaaactgcgatgtcgccattagttattttgtgacacaaaaacaaaatcgaaaacaaaagaaatagaaagaaaaagaaaaaaacaaaggctaaacaagattccattgtgcagatttaatgtttacaatataattacattcgctctttttgaccccagctgcacaacccacataattcgcataaaacaataacaattggcctaacatagatgccaaacacacttctgacataactgaatgctagggtaaaactccacacaggtttttgaacacgaactgccagtatacagctagccttgaaagtgatggaaaacattgtcatcggcacataaataaaaaaattatgaatcacgtgatcaacagcaaattatgtactcgtatatatgcaagcgcattccttatattgacactctttcattgggcatgaggctgtgtaaacataagctgtaacggtctcacggaacactgtgtagttgcattactgtcaggcagttatgttgcttaatataaaggcaaaggcatggcagaatcactttaaaacgtggccccacccgctgcaccctgtgcattttgtacgttactgcgaaatgactcaacgatgaaatgaaaaaattttgcttgaaattttgaaaaaaagatgtgcgggacgcattgttagctcattgaatacatcgtgatcgaatccaatcaatgtttaatttcatattaatacagaaactttttctcatactttcttcttcttttcattggtatattatctatatatcgtgttaattcctctttcgtcatttttagcatcgttataagagtaattaagaataactagaagagtctctctttatattcgtttattttatatatttaaccgctaaatttagtaaacaaaagaatctatcagaaatgagtgaatctccaatgttcgctgccaacggcatgccaaaggtaaatcaaggtgctgaagaagatgtcagaattttaggttacgacccattagcttctccagctctccttcaagtgcaaatcccagccacaccaacttctttggaaactgccaagagaggtagaagagaagctatagatattattaccggtaaagacgacagagttcttgtcattgtcggtccttgttccatccatgatctagaagccgctcaagaatacgctttgagattaaagaaattgtcagatgaattaaaaggtgatttatccatcattatgagagcatacttggagaagccaagaacaaccgtcggctggaaaggtctaattaatgaccctgatgttaacaacactttcaacatcaacaagggtttgcaatccgctagacaattgtttgtcaacttgacaaatatcggtttgccaattggttctgaaatgcttgataccatttctcctcaatacttggctgatttggtctccttcggtgccattggtgccagaaccaccgaatctcaactgcacagagaattggcctccggtttgtctttcccagttggtttcaagaacggtaccgatggtaccttaaatgttgctgtggatgcttgtcaagccgctgctcattctcaccatttcatgggtgttactaagcatggtgttgctgctatcaccactactaagggtaacgaacactgcttcgttattctaagaggtggtaaaaagggtaccaactacgacgctaagtccgttgcagaagctaaggctcaattgcctgccggttccaacggtctaatgattgactactctcacggtaactccaataaggatttcagaaaccaaccaaaggtcaatgacgttgtttgtgagcaaatcgctaacggtgaaaacgccattaccggtgtcatgattgaatcaaacatcaacgaaggtaaccaaggcatcccagccgaaggtaaagccggcttgaaatatggtgtttccatcactgatgcttgtataggttgggaaactactgaagacgtcttgaggaaattggctgctgctgtcagacaaagaagagaagttaacaagaaatagatgtttttttaatgatatatgtaacgtacattctttcctctaccactgccaattcggtattatttaattgtgtttagcgctatttactaattaactagaaactcaatttttaaaggcaaagctcgctgacctttcactgatttcgtggatgttatactatcagttactcttctgcaaaaaaaaattgagtcatatcgtagctttgggattatttttctctctctccacggctaattaggtgatcatgaaaaaatgaaaaattcatgagaaaagagtcagacatcgaaacatacataagttgatattcctttgatatcgacgactactcaatcaggttttaaaagaaaagaggcagctattgaagtagcagtatccagtttaggttttttaattatttataagtaaagaaaaagaaaaatcacctattgcgccgctcgcggaatacaattactaaattttatatatattctttaaaaatgcatctatacattcgtttttccacgtataccaaattcgaaaaaagttgttaaaccatcgttttcacgttttttaatttttttttggttctcttttttttttttttcaatatcaactttttttcaaacttcgtgttgcatttcctttatcgtaaattttcaatggatctctataatcttcgaagttcgaagaaaagaagaaaaaaagtattgaaaagttgaaacatcgattccgttttgctaacaaatagcactcagcatcctgcataaaattggtataagatatggatttcacaaaaccagaaactgttttaaatctacaaaatattagagatgaattagttagaatggaggattcgatcatcttcaaatttattgagaggtcgcatttcgccacatgtccttcagtttatgaggcaaaccatccaggtttagaaattccgaattttaaaggatctttcttggattgggctctttcaaatcttgaaattgcgcattctcgcatcagaagattcgaatcacctgatgaaactcccttctttcctgacaagattcagaaatcattcttaccgagcattaactacccacaaattttggcgccttatgccccagaagttaattacaatgataaaataaaaaaagtttatattgaaaagattataccattaatttcgaaaagagatggtgatgataagaataacttcagttctgttgccactagagatatagaatgtttgcaaagcttgagtaggagaatccactttggcaagtttgttgctgaagccaagttccaatcggatatcccgctatacacaaagctgatcaaaagtaaagatgtcgaggggataatgaagaatatcaccaattctgccgttgaagaaaagattctagaaagattaactaagaaggctgaagtctatggtgtggaccctaccaacgagtcaggtgaaagaaggattactccagaatatttggtaaaaatttataaggaaattgttatacctatcactaaggaagttgaggtggaatacttgctaagaaggttggaagagtaagcatgaaggctatcacggtaacaattccacattgccgagtgattactacgcatatataaacacacacactcaccatatatatgtacgcatagtagcatctattgaatataagaagatagagtttcaagttatacattatattgtactat

tttcttaaacg-3’

## 4.4 Sequence of the medium-module

5’-acttctcgtaggaacaatttcgggcccctgcgtgttcttctgaggttcatcttttacatttgcttctgctggataattttcaga

ggcaacaaggaaaaattagatggcaaaaagtcgtctttcaaggaaaaatccccaccatctttcgagatcccctgtaacttattggcaactgaaagaatgaaaaggaggaaaatacaaaatatactagaactgaaaaaaaaaaagtataaatagagacgatatatgccaatacttcacaatgttcgaatctattcttcatttgcagctattgtaaaataataaaacatcaagaacaaacaagctcaacttgtcttttctaagaacaaagaataaacacaaaaacaaaaagtttttttaattttaatcaaaaaatgactcaattcactgacattgataagctagccgtctccaccataagaattttggctgtggacaccgtatccaaggccaactcaggtcacccaggtgctccattgggtatggcaccagctgcacacgttctatggagtcaaatgcgcatgaacccaaccaacccagactggatcaacagagatagatttgtcttgtctaacggtcacgcggtcgctttgttgtattctatgctacatttgactggttacgatctgtctattgaagacttgaaacagttcagacagttgggttccagaacaccaggtcatcctgaatttgagttgccaggtgttgaagttactaccggtccattaggtcaaggtatctccaacgctgttggtatggccatggctcaagctaacctggctgccacttacaacaagccgggctttaccttgtctgacaactacacctatgttttcttgggtgacggttgtttgcaagaaggtatttcttcagaagcttcctccttggctggtcatttgaaattgggtaacttgattgccatctacgatgacaacaagatcactatcgatggtgctaccagtatctcattcgatgaagatgttgctaagagatacgaagcctacggttgggaagttttgtacgtagaaaatggtaacgaagatctagccggtattgccaaggctattgctcaagctaagttatccaaggacaaaccaactttgatcaaaatgaccacaaccattggttacggttccttgcatgccggctctcactctgtgcacggtgccccattgaaagcagatgatgttaaacaactaaagagcaaattcggtttcaacccagacaagtcctttgttgttccacaagaagtttacgaccactaccaaaagacaattttaaagccaggtgtcgaagccaacaacaagtggaacaagttgttcagcgaataccaaaagaaattcccagaattaggtgctgaattggctagaagattgagcggccaactacccgcaaattgggaatctaagttgccaacttacaccgccaaggactctgccgtggccactagaaaattatcagaaactgttcttgaggatgtttacaatcaattgccagagttgattggtggttctgccgatttaacaccttctaacttgaccagatggaaggaagcccttgacttccaacctccttcttccggttcaggtaactactctggtagatacattaggtacggtattagagaacacgctatgggtgccataatgaacggtatttcagctttcggtgccaactacaaaccatacggtggtactttcttgaacttcgtttcttatgctgctggtgccgttagattgtccgctttgtctggccacccagttatttgggttgctacacatgactctatcggtgtcggtgaagatggtccaacacatcaacctattgaaactttagcacacttcagatccctaccaaacattcaagtttggagaccagctgatggtaacgaagtttctgccgcctacaagaactctttagaatccaagcatactccaagtatcattgctttgtccagacaaaacttgccacaattggaaggtagctctattgaaagcgcttctaagggtggttacgtactacaagatgttgctaacccagatattattttagtggctactggttccgaagtgtctttgagtgttgaagctgctaagactttggccgcaaagaacatcaaggctcgtgttgtttctctaccagatttcttcacttttgacaaacaacccctagaatacagactatcagtcttaccagacaacgttccaatcatgtctgttgaagttttggctaccacatgttggggcaaatacgctcatcaatccttcggtattgacagatttggtgcctccggtaaggcaccagaagtcttcaagttcttcggtttcaccccagaaggtgttgctgaaagagctcaaaagaccattgcattctataagggtgacaagctaatttctcctttgaaaaaagctttctaagcggatctcttatgtctttacgatttatagttttcattatcaagtatgcctatattagtatatagcatctttagatgacagtgttcgaagtttcacgaataaaagataatattctactttttgctcccaccgcgtttgctagcacgagtgaacaccatccctcgcctgtgagttgtacccattcctctaaactgtagacatggtagcttcagcagtgttcgttatgtacggcatcctccaacaaacagtcggttatagtttgtcctgctcctctgaatcgagtccctcgatatttctcatccacacaccatagcttcaaaatgtttctactccttttttactcttccagattttctcggactccgcgcatcgccgtaccacttcaaaacacccaagcacagcatactaaatttcccctctttcttcctctagggtgtcgttaattacccgtactaaaggtttggaaaagaaaaaagagaccgcctcgtttctttttcttcgtcgaaaaaggcaataaaaatttttatcacgtttctttttcttgaaaatttttttttttgatttttttctctttcgatgacctcccattgatatttaagttaataaacggtcttcaatttctcaagtttcagtttcatttttcttgttctattacaactttttttacttcttgctcattagaaagaaagcatagcaatctaatctaagatggctgccggtgtcccaaaaattgatgcgttagaatctttgggcaatcctttggaggatgccaagagagctgcagcatacagagcagttgatgaaaatttaaaatttgatgatcacaaaattattggaattggtagtggtagcacagtggtttatgttgccgaaagaattggacaatatttgcatgaccctaaattttatgaagtagcgtctaaattcatttgcattccaacaggattccaatcaagaaacttgattttggataacaagttgcaattaggctccattgaacagtatcctcgcattgatatagcgtttgacggtgctgatgaagtggatgagaatttacaattaattaaaggtggtggtgcttgtctatttcaagaaaaattggttagtactagtgctaaaaccttcattgtcgttgctgattcaagaaaaaagtcaccaaaacatttaggtaagaactggaggcaaggtgttcccattgaaattgtaccttcctcatacgtgagggtcaagaatgatctattagaacaattgcatgctgaaaaagttgacatcagacaaggaggttctgctaaagcaggtcctgttgtaactgacaataataacttcattatcgatgcggatttcggtgaaatttccgatccaagaaaattgcatagagaaatcaaactgttagtgggcgtggtggaaacaggtttattcatcgacaacgcttcaaaagcctacttcggtaattctgacggtagtgttgaagttaccgaaaagtgaattgaattgaattgaaatcgatagatcaatttttttcttttctctttccccatcctttacgctaaaataatagtttattttattttttgaatattttttatttatatacgtatatatagactattatttatcttttaatgattattaagatttttattaaaaaaaaattcgctcctcttttaatgcctttatgcagtttttttttcccattcgatatttctatgttcgggttcagcgtattttaagtttaataactcgaaaattctgcgttggatacttcatgctatttatagacgcgcgtgtcggaatcagcacgcgcaagaaccaaatgggaaaatcggaatgggtccagaactgctttgagtgctggctattggcgtctgatttccgttttgggaatcctttgccgcgcgcccctctcaaaactccgcacaagtcccagaaagcgggaaagaaataaaacgccaccaaaaaaaaaaaaataaaagccaatcctcgaagcgtgggtggtaggccctggattatcccgtacaagtatttctcaggagtaaaaaaaccgtttgttttggaattccccatttcgcggccacctacgccgctatctttgcaacaactatctgcgataactcagcaaattttgcatattcgtgttgcagtattgcgataatgggagtcttacttccaacataacggcagaaagaaatgtgagaaaattttgcatcctttgcctccgttcaagtatataaagtcggcatgcttgataatctttctttccatcctacattgttctaattattcttattctcctttattctttcctaacataccaagaaattaatcttctgtcattcgcttaaacactatatcaataatggcacctgttacaattgaaaagttcgtaaatcaagaagaacgacaccttgtttccaaccgatcagcaacaattccgtttggtgaatacatatttaaaagattgttgtccatcgatacgaaatcagttttcggtgttcctggtgacttcaacttatctctattagaatatctctattcacctagtgttgaatcagctggcctaagatgggtcggcacgtgtaatgaactgaacgccgcttatgcggccgacggatattcccgttactctaataagattggctgtttaataaccacgtatggcgttggtgaattaagcgccttgaacggtatagccggttcgttcgctgaaaatgtcaaagttttgcacattgttggtgtggccaagtccatagattcgcgttcaagtaactttagtgatcggaacctacatcatttggtcccacagctacatgattcaaattttaaagggccaaatcataaagtatatcatgatatggtaaaagatagagtcgcttgctcggtagcctacttggaggatattgaaactgcatgtgaccaagtcgataatgttatccgcgatatttacaagtattctaaacctggttatatttttgttcctgcagattttgcggatatgtctgttacatgtgataatttggttaatgttccacgtatatctcaacaagattgtatagtatacccttctgaaaaccaattgtctgacataatcaacaagattactagttggatatattccagtaaaacacctgcgatccttggagacgtactgactgataggtatggtgtgagtaactttttgaacaagcttatctgcaaaactgggatttggaatttttccactgttatgggaaaatctgtaattgatgagtcaaacccaacttatatgggtcaatataatggtaaagaaggtttaaaacaagtctatgaacattttgaactgtgcgacttggtcttgcattttggagtcgacatcaatgaaattaataatgggcattatacttttacttataaaccaaatgctaaaatcattcaatttcatccgaattatattcgccttgtggacactaggcagggcaatgagcaaatgttcaaaggaatcaattttgcccctattttaaaagaactatacaagcgcattgacgtttctaaactttctttgcaatatgattcaaatgtaactcaatatacgaacgaaacaatgcggttagaagatcctaccaatggacaatcaagcattattacacaagttcacttacaaaagacgatgcctaaatttttgaaccctggtgatgttgtcgtttgtgaaacaggctcttttcaattctctgttcgtgatttcgcgtttccttcgcaattaaaatatatatcgcaaggatttttcctttccattggcatggcccttcctgccgccctaggtgttggaattgccatgcaagaccactcaaacgctcacatcaatggtggcaacgtaaaagaggactataagccaagattaattttgtttgaaggtgacggtgcagcacagatgacaatccaagaactgagcaccattctgaagtgcaatattccactagaagttatcatttggaacaataacggctacactattgaaagagccatcatgggccctaccaggtcgtataacgacgttatgtcttggaaatggaccaaactatttgaagcattcggagacttcgacggaaagtatactaatagcactctcattcaatgtccctctaaattagcactgaaattggaggagcttaagaattcaaacaaaagaagcgggatagaacttttagaagtcaaattaggcgaattggatttccccgaacagctaaagtgcatggttgaagcagcggcacttaaaagaaataaaaaataggcgaatttcttatgatttatgatttttattattaaataagttataaaaaaaataagtgtatacaaattttaaagtgactcttaggttttaaaacgaaaattcttattcttgagtaactctttcctgtaggtcaggttgctttctcaggtatagcatgaggtcgctcttattgaccacacctctaccggtcaggcatgaacgcatcacagacaaaatcttcttgacaaacgtcacaattgatccctccccatccgttatcacaatgacaggtgtcattttgtgctcttatgggacgatccttattaccgctttcatccggtgatagaccgccacagaggggcagagagcaatcatcacctgcaaacccttctatacactcacatctaccagtgtacgaattgcattcagaaaactgtttgcattcaaaaataggtagcatacaattaaaacatggcgggcacgtatcattgcccttatcttgtgcagttagacgcgaatttttcgaagaagtaccttcaaagaatggggtctcatcttgttttgcaagtaccactgagcaggataataatagaaatgataatatactatagtagagataacgtcgatgacttcccatactgtaattgcttttagttgtgtatttttagtgtgcaagtttctgtaaatcgattaatttttttttctttcctctttttattaaccttaatttttattttagattcctgacttcaactcaagacgcacagatattataacatctgcacaataggcatttgcaagaattactcgtgagtaaggaaagagtgaggaactatcgcatacctgcatttaaagatgccgatttgggcgcgaatcctttattttggcttcaccctcatactattatcagggccagaaaaaggaagtgtttccctccttcttgaattgatgttaccctcataaagcacgtggcctcttatcgagaaagaaattaccgtcgctcgtgatttgtttgcaaaaagaacaaaactgaaaaaacccagacacgctcgacttcctgtcttcctattgattgcagcttccaatttcgtcacacaacaaggtcctagcgacggctcacaggttttgtaacaagcaatcgaaggttctggaatggcgggaaagggtttagtaccacatgctatgatgcccactgtgatctccagagcaaagttcgttcgatcgtactgttactctctctctttcaaacagaattgtccgaatcgtgtgacaacaacagcctgttctcacacactcttttcttctaaccaagggggtggtttagtttagtagaacctcgtgaaacttacatttacatatatataaacttgcataaattggtcaatgcaagaaatacatatttggtcttttctaattcgtagtttttcaagttcttagatgctttctttttctcttttttacagatcatcaaggaagtaattatctactttttacaacaaatataaaacaatgtcaacgtttgggaaactgttccgcgtcaccacatatggtgaatcgcattgtaagtctgtcggttgcattgtcgacggtgttcctccaggaatgtcattaaccgaagctgacattcagccacaattgaccagaagaagaccgggtcaatctaagctatcgacccctagagacgaaaaggatagagtggaaatccagtccggtaccgagttcggcaagactctaggtacacccatcgccatgatgatcaaaaacgaggaccaaagacctcacgactactccgacatggacaagttccctagaccttcccatgcggacttcacgtactcggaaaagtacggtatcaaggcctcctctggtggtggcagagcttctgctagagaaacgattggccgtgtcgcttcaggtgccattgctgagaagttcttagctcagaactctaatgtcgagatcgtagcctttgtgacacaaatcggggaaatcaagatgaacagagactctttcgatcctgaatttcagcatctgttgaacaccatcaccagggaaaaagtggactcaatgggtcctatcagatgtccagacgcctccgttgctggtttgatggtcaaggaaatcgaaaagtacagaggcaacaaggactctatcggtggtgtcgtcacttgtgtcgtgagaaacttgcctaccggtctcggtgagccatgctttgacaagttggaagccatgttggctcatgctatgttgtccattccagcatccaagggtttcgaaattggctcaggttttcagggtgtctctgttccagggtccaagcacaatgacccattttactttgaaaaagaaacaaacagattaagaacaaagaccaacaattcaggtggtgtacaaggtggtatctctaatggtgagaacatctatttctctgtcccattcaagtcagtggccactatctctcaagaacaaaaaaccgccacttacgatggtgaagaaggtatcttagccgctaagggtagacatgaccctgctgtcactccaagagctattcctattgtggaagccatgaccgctctggtgttggctgacgcgcttttgatccaaaaggcaagagatttctccagatccgtggttcattaagtgaatttactttaaatcttgcatttaaataaattttctttttatagctttatgacttagtttcaatttatatactattttaatgacattttcgattcattgattgaaagctttgtgttttttcttgatgcgctattgcattgttcttgtctttttcgccacatgtaatatctgtagtagatacctgatacattgtggatgctgagtgaaattttagttaataatggaggcgctcttaataattttggggatattggcttttttttttaaagtttacaaatgaattttttccgccaggataacgattctgaagttactcttagcgttcctatcggtacagccatcaaatcatgcctataaatcatgcctatatttgcgtgcagtcagtatcatctacatgaaaaaaactcccgcaatttcttatagaatacgttgaaaattaaatgtacgcgccaagataagataacat

atatctagatgcagtaatatacacagattcc-3’

## 4.5 Sequence of the large-module

5’-acttctcgtaggaacaatttcgggcccctgcgtgttcttctgaggttcatcttttacatttgcttctgctggataattttcaga

ggcaacaaggaaaaattagatggcaaaaagtcgtctttcaaggaaaaatccccaccatctttcgagatcccctgtaacttattggcaactgaaagaatgaaaaggaggaaaatacaaaatatactagaactgaaaaaaaaaaagtataaatagagacgatatatgccaatacttcacaatgttcgaatctattcttcatttgcagctattgtaaaataataaaacatcaagaacaaacaagctcaacttgtcttttctaagaacaaagaataaacacaaaaacaaaaagtttttttaattttaatcaaaaaatgactcaattcactgacattgataagctagccgtctccaccataagaattttggctgtggacaccgtatccaaggccaactcaggtcacccaggtgctccattgggtatggcaccagctgcacacgttctatggagtcaaatgcgcatgaacccaaccaacccagactggatcaacagagatagatttgtcttgtctaacggtcacgcggtcgctttgttgtattctatgctacatttgactggttacgatctgtctattgaagacttgaaacagttcagacagttgggttccagaacaccaggtcatcctgaatttgagttgccaggtgttgaagttactaccggtccattaggtcaaggtatctccaacgctgttggtatggccatggctcaagctaacctggctgccacttacaacaagccgggctttaccttgtctgacaactacacctatgttttcttgggtgacggttgtttgcaagaaggtatttcttcagaagcttcctccttggctggtcatttgaaattgggtaacttgattgccatctacgatgacaacaagatcactatcgatggtgctaccagtatctcattcgatgaagatgttgctaagagatacgaagcctacggttgggaagttttgtacgtagaaaatggtaacgaagatctagccggtattgccaaggctattgctcaagctaagttatccaaggacaaaccaactttgatcaaaatgaccacaaccattggttacggttccttgcatgccggctctcactctgtgcacggtgccccattgaaagcagatgatgttaaacaactaaagagcaaattcggtttcaacccagacaagtcctttgttgttccacaagaagtttacgaccactaccaaaagacaattttaaagccaggtgtcgaagccaacaacaagtggaacaagttgttcagcgaataccaaaagaaattcccagaattaggtgctgaattggctagaagattgagcggccaactacccgcaaattgggaatctaagttgccaacttacaccgccaaggactctgccgtggccactagaaaattatcagaaactgttcttgaggatgtttacaatcaattgccagagttgattggtggttctgccgatttaacaccttctaacttgaccagatggaaggaagcccttgacttccaacctccttcttccggttcaggtaactactctggtagatacattaggtacggtattagagaacacgctatgggtgccataatgaacggtatttcagctttcggtgccaactacaaaccatacggtggtactttcttgaacttcgtttcttatgctgctggtgccgttagattgtccgctttgtctggccacccagttatttgggttgctacacatgactctatcggtgtcggtgaagatggtccaacacatcaacctattgaaactttagcacacttcagatccctaccaaacattcaagtttggagaccagctgatggtaacgaagtttctgccgcctacaagaactctttagaatccaagcatactccaagtatcattgctttgtccagacaaaacttgccacaattggaaggtagctctattgaaagcgcttctaagggtggttacgtactacaagatgttgctaacccagatattattttagtggctactggttccgaagtgtctttgagtgttgaagctgctaagactttggccgcaaagaacatcaaggctcgtgttgtttctctaccagatttcttcacttttgacaaacaacccctagaatacagactatcagtcttaccagacaacgttccaatcatgtctgttgaagttttggctaccacatgttggggcaaatacgctcatcaatccttcggtattgacagatttggtgcctccggtaaggcaccagaagtcttcaagttcttcggtttcaccccagaaggtgttgctgaaagagctcaaaagaccattgcattctataagggtgacaagctaatttctcctttgaaaaaagctttctaagcggatctcttatgtctttacgatttatagttttcattatcaagtatgcctatattagtatatagcatctttagatgacagtgttcgaagtttcacgaataaaagataatattctactttttgctcccaccgcgtttgctagcacgagtgaacaccatccctcgcctgtgagttgtacccattcctctaaactgtagacatggtagcttcagcagtgttcgttatgtacggcatcctccaacaaacagtcggttatagtttgtcctgctcctctgaatcgagtccctcgatatttctcatccacacaccatagcttcaaaatgtttctactccttttttactcttccagattttctcggactccgcgcatcgccgtaccacttcaaaacacccaagcacagcatactaaatttcccctctttcttcctctagggtgtcgttaattacccgtactaaaggtttggaaaagaaaaaagagaccgcctcgtttctttttcttcgtcgaaaaaggcaataaaaatttttatcacgtttctttttcttgaaaatttttttttttgatttttttctctttcgatgacctcccattgatatttaagttaataaacggtcttcaatttctcaagtttcagtttcatttttcttgttctattacaactttttttacttcttgctcattagaaagaaagcatagcaatctaatctaagatggctgccggtgtcccaaaaattgatgcgttagaatctttgggcaatcctttggaggatgccaagagagctgcagcatacagagcagttgatgaaaatttaaaatttgatgatcacaaaattattggaattggtagtggtagcacagtggtttatgttgccgaaagaattggacaatatttgcatgaccctaaattttatgaagtagcgtctaaattcatttgcattccaacaggattccaatcaagaaacttgattttggataacaagttgcaattaggctccattgaacagtatcctcgcattgatatagcgtttgacggtgctgatgaagtggatgagaatttacaattaattaaaggtggtggtgcttgtctatttcaagaaaaattggttagtactagtgctaaaaccttcattgtcgttgctgattcaagaaaaaagtcaccaaaacatttaggtaagaactggaggcaaggtgttcccattgaaattgtaccttcctcatacgtgagggtcaagaatgatctattagaacaattgcatgctgaaaaagttgacatcagacaaggaggttctgctaaagcaggtcctgttgtaactgacaataataacttcattatcgatgcggatttcggtgaaatttccgatccaagaaaattgcatagagaaatcaaactgttagtgggcgtggtggaaacaggtttattcatcgacaacgcttcaaaagcctacttcggtaattctgacggtagtgttgaagttaccgaaaagtgaattgaattgaattgaaatcgatagatcaatttttttcttttctctttccccatcctttacgctaaaataatagtttattttattttttgaatattttttatttatatacgtatatatagactattatttatcttttaatgattattaagatttttattaaaaaaaaattcgctcctcttttaatgcctttatgcagtttttttttcccattcgatatttctatgttcgggttcagcgtattttaagtttaataactcgaaaattctgcgttggatacttcatgctatttatagacgcgcgtgtcggaatcagcacgcgcaagaaccaaatgggaaaatcggaatgggtccagaactgctttgagtgctggctattggcgtctgatttccgttttgggaatcctttgccgcgcgcccctctcaaaactccgcacaagtcccagaaagcgggaaagaaataaaacgccaccaaaaaaaaaaaaataaaagccaatcctcgaagcgtgggtggtaggccctggattatcccgtacaagtatttctcaggagtaaaaaaaccgtttgttttggaattccccatttcgcggccacctacgccgctatctttgcaacaactatctgcgataactcagcaaattttgcatattcgtgttgcagtattgcgataatgggagtcttacttccaacataacggcagaaagaaatgtgagaaaattttgcatcctttgcctccgttcaagtatataaagtcggcatgcttgataatctttctttccatcctacattgttctaattattcttattctcctttattctttcctaacataccaagaaattaatcttctgtcattcgcttaaacactatatcaataatggcacctgttacaattgaaaagttcgtaaatcaagaagaacgacaccttgtttccaaccgatcagcaacaattccgtttggtgaatacatatttaaaagattgttgtccatcgatacgaaatcagttttcggtgttcctggtgacttcaacttatctctattagaatatctctattcacctagtgttgaatcagctggcctaagatgggtcggcacgtgtaatgaactgaacgccgcttatgcggccgacggatattcccgttactctaataagattggctgtttaataaccacgtatggcgttggtgaattaagcgccttgaacggtatagccggttcgttcgctgaaaatgtcaaagttttgcacattgttggtgtggccaagtccatagattcgcgttcaagtaactttagtgatcggaacctacatcatttggtcccacagctacatgattcaaattttaaagggccaaatcataaagtatatcatgatatggtaaaagatagagtcgcttgctcggtagcctacttggaggatattgaaactgcatgtgaccaagtcgataatgttatccgcgatatttacaagtattctaaacctggttatatttttgttcctgcagattttgcggatatgtctgttacatgtgataatttggttaatgttccacgtatatctcaacaagattgtatagtatacccttctgaaaaccaattgtctgacataatcaacaagattactagttggatatattccagtaaaacacctgcgatccttggagacgtactgactgataggtatggtgtgagtaactttttgaacaagcttatctgcaaaactgggatttggaatttttccactgttatgggaaaatctgtaattgatgagtcaaacccaacttatatgggtcaatataatggtaaagaaggtttaaaacaagtctatgaacattttgaactgtgcgacttggtcttgcattttggagtcgacatcaatgaaattaataatgggcattatacttttacttataaaccaaatgctaaaatcattcaatttcatccgaattatattcgccttgtggacactaggcagggcaatgagcaaatgttcaaaggaatcaattttgcccctattttaaaagaactatacaagcgcattgacgtttctaaactttctttgcaatatgattcaaatgtaactcaatatacgaacgaaacaatgcggttagaagatcctaccaatggacaatcaagcattattacacaagttcacttacaaaagacgatgcctaaatttttgaaccctggtgatgttgtcgtttgtgaaacaggctcttttcaattctctgttcgtgatttcgcgtttccttcgcaattaaaatatatatcgcaaggatttttcctttccattggcatggcccttcctgccgccctaggtgttggaattgccatgcaagaccactcaaacgctcacatcaatggtggcaacgtaaaagaggactataagccaagattaattttgtttgaaggtgacggtgcagcacagatgacaatccaagaactgagcaccattctgaagtgcaatattccactagaagttatcatttggaacaataacggctacactattgaaagagccatcatgggccctaccaggtcgtataacgacgttatgtcttggaaatggaccaaactatttgaagcattcggagacttcgacggaaagtatactaatagcactctcattcaatgtccctctaaattagcactgaaattggaggagcttaagaattcaaacaaaagaagcgggatagaacttttagaagtcaaattaggcgaattggatttccccgaacagctaaagtgcatggttgaagcagcggcacttaaaagaaataaaaaataggcgaatttcttatgatttatgatttttattattaaataagttataaaaaaaataagtgtatacaaattttaaagtgactcttaggttttaaaacgaaaattcttattcttgagtaactctttcctgtaggtcaggttgctttctcaggtatagcatgaggtcgctcttattgaccacacctctaccggtcaggcatgaacgcatcacagacaaaatcttcttgacaaacgtcacaattgatccctccccatccgttatcacaatgacaggtgtcattttgtgctcttatgggacgatccttattaccgctttcatccggtgatagaccgccacagaggggcagagagcaatcatcacctgcaaacccttctatacactcacatctaccagtgtacgaattgcattcagaaaactgtttgcattcaaaaataggtagcatacaattaaaacatggcgggcacgtatcattgcccttatcttgtgcagttagacgcgaatttttcgaagaagtaccttcaaagaatggggtctcatcttgttttgcaagtaccactgagcaggataataatagaaatgataatatactatagtagagataacgtcgatgacttcccatactgtaattgcttttagttgtgtatttttagtgtgcaagtttctgtaaatcgattaatttttttttctttcctctttttattaaccttaatttttattttagattcctgacttcaactcaagacgcacagatattataacatctgcacaataggcatttgcaagaattactcgtgagtaaggaaagagtgaggaactatcgcatacctgcatttaaagatgccgatttgggcgcgaatcctttattttggcttcaccctcatactattatcagggccagaaaaaggaagtgtttccctccttcttgaattgatgttaccctcataaagcacgtggcctcttatcgagaaagaaattaccgtcgctcgtgatttgtttgcaaaaagaacaaaactgaaaaaacccagacacgctcgacttcctgtcttcctattgattgcagcttccaatttcgtcacacaacaaggtcctagcgacggctcacaggttttgtaacaagcaatcgaaggttctggaatggcgggaaagggtttagtaccacatgctatgatgcccactgtgatctccagagcaaagttcgttcgatcgtactgttactctctctctttcaaacagaattgtccgaatcgtgtgacaacaacagcctgttctcacacactcttttcttctaaccaagggggtggtttagtttagtagaacctcgtgaaacttacatttacatatatataaacttgcataaattggtcaatgcaagaaatacatatttggtcttttctaattcgtagtttttcaagttcttagatgctttctttttctcttttttacagatcatcaaggaagtaattatctactttttacaacaaatataaaacaatgtcaacgtttgggaaactgttccgcgtcaccacatatggtgaatcgcattgtaagtctgtcggttgcattgtcgacggtgttcctccaggaatgtcattaaccgaagctgacattcagccacaattgaccagaagaagaccgggtcaatctaagctatcgacccctagagacgaaaaggatagagtggaaatccagtccggtaccgagttcggcaagactctaggtacacccatcgccatgatgatcaaaaacgaggaccaaagacctcacgactactccgacatggacaagttccctagaccttcccatgcggacttcacgtactcggaaaagtacggtatcaaggcctcctctggtggtggcagagcttctgctagagaaacgattggccgtgtcgcttcaggtgccattgctgagaagttcttagctcagaactctaatgtcgagatcgtagcctttgtgacacaaatcggggaaatcaagatgaacagagactctttcgatcctgaatttcagcatctgttgaacaccatcaccagggaaaaagtggactcaatgggtcctatcagatgtccagacgcctccgttgctggtttgatggtcaaggaaatcgaaaagtacagaggcaacaaggactctatcggtggtgtcgtcacttgtgtcgtgagaaacttgcctaccggtctcggtgagccatgctttgacaagttggaagccatgttggctcatgctatgttgtccattccagcatccaagggtttcgaaattggctcaggttttcagggtgtctctgttccagggtccaagcacaatgacccattttactttgaaaaagaaacaaacagattaagaacaaagaccaacaattcaggtggtgtacaaggtggtatctctaatggtgagaacatctatttctctgtcccattcaagtcagtggccactatctctcaagaacaaaaaaccgccacttacgatggtgaagaaggtatcttagccgctaagggtagacatgaccctgctgtcactccaagagctattcctattgtggaagccatgaccgctctggtgttggctgacgcgcttttgatccaaaaggcaagagatttctccagatccgtggttcattaagtgaatttactttaaatcttgcatttaaataaattttctttttatagctttatgacttagtttcaatttatatactattttaatgacattttcgattcattgattgaaagctttgtgttttttcttgatgcgctattgcattgttcttgtctttttcgccacatgtaatatctgtagtagatacctgatacattgtggatgctgagtgaaattttagttaataatggaggcgctcttaataattttggggatattggcttttttttttaaagtttacaaatgaattttttccgccaggataacgattctgaagttactcttagcgttcctatcggtacagccatcaaatcatgcctataaatcatgcctatatttgcgtgcagtcagtatcatctacatgaaaaaaactcccgcaatttcttatagaatacgttgaaaattaaatgtacgcgccaagataagataacatatatctagatgcagtaatatacacagattcctagtcaaacaaaaaagggaaattctatttgtctgattttgttgcaagataacaatactggcaaacatgggcttgagtgcttaggaagaaatcttgaggtagcagtaacttctttttattaaacagtatgcagccgcccaaaaaactgcgatgtcgccattagttattttgtgacacaaaaacaaaatcgaaaacaaaagaaatagaaagaaaaagaaaaaaacaaaggctaaacaagattccattgtgcagatttaatgtttacaatataattacattcgctctttttgaccccagctgcacaacccacataattcgcataaaacaataacaattggcctaacatagatgccaaacacacttctgacataactgaatgctagggtaaaactccacacaggtttttgaacacgaactgccagtatacagctagccttgaaagtgatggaaaacattgtcatcggcacataaataaaaaaattatgaatcacgtgatcaacagcaaattatgtactcgtatatatgcaagcgcattccttatattgacactctttcattgggcatgaggctgtgtaaacataagctgtaacggtctcacggaacactgtgtagttgcattactgtcaggcagttatgttgcttaatataaaggcaaaggcatggcagaatcactttaaaacgtggccccacccgctgcaccctgtgcattttgtacgttactgcgaaatgactcaacgatgaaatgaaaaaattttgcttgaaattttgaaaaaaagatgtgcgggacgcattgttagctcattgaatacatcgtgatcgaatccaatcaatgtttaatttcatattaatacagaaactttttctcatactttcttcttcttttcattggtatattatctatatatcgtgttaattcctctttcgtcatttttagcatcgttataagagtaattaagaataactagaagagtctctctttatattcgtttattttatatatttaaccgctaaatttagtaaacaaaagaatctatcagaaatgagtgaatctccaatgttcgctgccaacggcatgccaaaggtaaatcaaggtgctgaagaagatgtcagaattttaggttacgacccattagcttctccagctctccttcaagtgcaaatcccagccacaccaacttctttggaaactgccaagagaggtagaagagaagctatagatattattaccggtaaagacgacagagttcttgtcattgtcggtccttgttccatccatgatctagaagccgctcaagaatacgctttgagattaaagaaattgtcagatgaattaaaaggtgatttatccatcattatgagagcatacttggagaagccaagaacaaccgtcggctggaaaggtctaattaatgaccctgatgttaacaacactttcaacatcaacaagggtttgcaatccgctagacaattgtttgtcaacttgacaaatatcggtttgccaattggttctgaaatgcttgataccatttctcctcaatacttggctgatttggtctccttcggtgccattggtgccagaaccaccgaatctcaactgcacagagaattggcctccggtttgtctttcccagttggtttcaagaacggtaccgatggtaccttaaatgttgctgtggatgcttgtcaagccgctgctcattctcaccatttcatgggtgttactaagcatggtgttgctgctatcaccactactaagggtaacgaacactgcttcgttattctaagaggtggtaaaaagggtaccaactacgacgctaagtccgttgcagaagctaaggctcaattgcctgccggttccaacggtctaatgattgactactctcacggtaactccaataaggatttcagaaaccaaccaaaggtcaatgacgttgtttgtgagcaaatcgctaacggtgaaaacgccattaccggtgtcatgattgaatcaaacatcaacgaaggtaaccaaggcatcccagccgaaggtaaagccggcttgaaatatggtgtttccatcactgatgcttgtataggttgggaaactactgaagacgtcttgaggaaattggctgctgctgtcagacaaagaagagaagttaacaagaaatagatgtttttttaatgatatatgtaacgtacattctttcctctaccactgccaattcggtattatttaattgtgtttagcgctatttactaattaactagaaactcaatttttaaaggcaaagctcgctgacctttcactgatttcgtggatgttatactatcagttactcttctgcaaaaaaaaattgagtcatatcgtagctttgggattatttttctctctctccacggctaattaggtgatcatgaaaaaatgaaaaattcatgagaaaagagtcagacatcgaaacatacataagttgatattcctttgatatcgacgactactcaatcaggttttaaaagaaaagaggcagctattgaagtagcagtatccagtttaggttttttaattatttataagtaaagaaaaagaaaaatcacctattgcgccgctcgcggaatacaattactaaattttatatatattctttaaaaatgcatctatacattcgtttttccacgtataccaaattcgaaaaaagttgttaaaccatcgttttcacgttttttaatttttttttggttctcttttttttttttttcaatatcaactttttttcaaacttcgtgttgcatttcctttatcgtaaattttcaatggatctctataatcttcgaagttcgaagaaaagaagaaaaaaagtattgaaaagttgaaacatcgattccgttttgctaacaaatagcactcagcatcctgcataaaattggtataagatatggatttcacaaaaccagaaactgttttaaatctacaaaatattagagatgaattagttagaatggaggattcgatcatcttcaaatttattgagaggtcgcatttcgccacatgtccttcagtttatgaggcaaaccatccaggtttagaaattccgaattttaaaggatctttcttggattgggctctttcaaatcttgaaattgcgcattctcgcatcagaagattcgaatcacctgatgaaactcccttctttcctgacaagattcagaaatcattcttaccgagcattaactacccacaaattttggcgccttatgccccagaagttaattacaatgataaaataaaaaaagtttatattgaaaagattataccattaatttcgaaaagagatggtgatgataagaataacttcagttctgttgccactagagatatagaatgtttgcaaagcttgagtaggagaatccactttggcaagtttgttgctgaagccaagttccaatcggatatcccgctatacacaaagctgatcaaaagtaaagatgtcgaggggataatgaagaatatcaccaattctgccgttgaagaaaagattctagaaagattaactaagaaggctgaagtctatggtgtggaccctaccaacgagtcaggtgaaagaaggattactccagaatatttggtaaaaatttataaggaaattgttatacctatcactaaggaagttgaggtggaatacttgctaagaaggttggaagagtaagcatgaaggctatcacggtaacaattccacattgccgagtgattactacgcatatataaacacacacactcaccatatatatgtacgcatagtagcatctattgaatataagaagata

gagtttcaagttatacattatattgtactattttcttaaacg-3’
